# Lamin B1 safeguards the B cell genome and shapes lymphoma outcome

**DOI:** 10.1002/hem3.70387

**Published:** 2026-06-07

**Authors:** Filip Filipsky, Katarina B. Chapman, Johannes Bloehdorn, Oscar Maiques, Abigail Lee, Andrew Clear, Jun Wang, John Gribben, Michael Hausmann, Christoph Cremer, Andrejs Braun, Marta C. Sallan, Tanya Klymenko

**Affiliations:** ^1^ Centre for Haemato‐Oncology, Barts Cancer Institute, Queen Mary University of London, 3rd Floor John Vane Science Centre, Charterhouse Square London UK; ^2^ Department of Biomedicine University Hospital and University of Basel Basel Switzerland; ^3^ Kirchhoff‐Institute for Physics, Heidelberg University, Heidelberg Germany; ^4^ Centre for Cancer Immunology, Faculty of Medicine University of Southampton Southampton UK; ^5^ Cancer Biomarkers & Biotherapeutics, Barts Cancer Institute, Queen Mary University of London London UK; ^6^ Department of Cellular Pathology Barts Health NHS Trust, Royal London Hospital London UK; ^7^ Institute of Molecular Biology Mainz Germany; ^8^ School of Biological and Behavioural Sciences, Queen Mary University of London London UK; ^9^ Present address: Department of Optical Nanoscopy Max Planck Institute for Medical Research Heidelberg 69120 Germany

## Abstract

Lamin B1 is a structural component of the nuclear lamina that participates in genome organization and transcriptional control. During adaptive immune responses, B lymphocytes in germinal centers (GCs) undergo clonal expansion and programmed DNA damage at immunoglobulin loci, while simultaneously downregulating Lamin B1. Likewise, Lamin B1 downregulation has been observed in GC‐derived lymphomas and myeloid malignancies, yet the functional consequences of Lamin B1 loss during B cell development remain poorly understood. Here, we used in vivo and in vitro B cell models of conditional hypomorphic Lamin B1 expression, which showed elevated DNA damage and disrupted transcriptional profiles. Using sBLISS (in situ labeling and sequencing of double‐strand breaks), we identified nonrandom double‐strand break hotspots in both mouse and human GC B cells depleted of Lamin B1. These breaks are preferentially located near transcriptional start sites (TSSs) and regulatory elements that control translation and mRNA fate, suggesting Lamin B1 has a role in protecting regulatory genomic regions. Moreover, low LMNB1 expression is associated with poor clinical outcomes in patients with diffuse large B‐cell lymphoma (DLBCL). Together, this study reveals a crucial role for Lamin B1 in preserving genomic stability in B cells, underscoring its impact on the pathogenesis of B cell‐derived malignancies.

## INTRODUCTION

The nuclear lamina (NL) is a structural component of the metazoan nucleus. It is composed of type V intermediate filaments (Lamin A/C and Lamin B1/B2) that shape nuclear architecture and genome organization through interactions with the inner nuclear membrane and chromatin.^1^, ^2^ Beyond structural role, NL contributes to a range of biological processes, including genome organization, gene transcription, and mechanical support by stabilizing the nuclear envelope.^3^, ^4^, ^5^

Importantly, A‐ and B‐type lamin networks are not redundant; differences in their structural properties confer distinct contributions to NL organization and function.^6^, ^7^, ^8^ Reflecting this functional specificity, pathogenic LMNA mutations cause laminopathies such as Hutchinson–Gilford progeria syndrome,^3^ whereas increased LMNB1 overexpression is causally linked to autosomal dominant leukodystrophy (ADLD),^5^ underscoring how disruption of lamina homeostasis can drive profound tissue dysfunction. In hematological contexts, lamina perturbations have also been associated with disease phenotypes, including a role for lamin B1 in acquired Pelger–Huët anomaly (pseudo‐PHA),^9^ alongside reports of aberrant lamin A/C expression in cancer, with early observations in Hodgkin lymphoma.^10^, ^11^, ^12^

Whereas A‐type lamins are often discussed in the context of laminopathies, Lamin B1 has emerged as a key organizer of peripheral heterochromatin and replication–transcription landscapes. Lamin B1 forms genomic regions known as lamina‐associated domains (LADs).^13^ The removal of Lamin B1 from the nuclear periphery drives major conformational changes in LADs during differentiation, leading to chromatin reorganization and differential gene expression.^14^, ^15^

Constant expression of Lamin B1 within the nucleus likely reflects low protein turnover and high stability mediated by extensive post‐translational modifications.^16^, ^17^, ^18^ In recent years, several studies have shown that the abnormal expression of LMNB1, a classical biomarker of cellular senescence, is strongly correlated with malignant transformation and influences clinical outcomes in cancer.^19^, ^20^, ^21^, ^22^ The potential clinical predictive value of Lamin B1 is disease‐dependent, and *LMNB1* reduction can be linked to either favorable^23^, ^24^, ^25^ or poor clinical outcomes in chronic lymphocytic leukemia (CLL) patients.^9^, ^26^, ^27^

Within the physiological context of B‐cell development, we have previously demonstrated that Lamin B1 has a direct role in safeguarding the genome from point mutations in GC B cells.^26^ In cell‐mediated immune responses, Lamin B1 is reduced from the nuclear periphery in B cells transiting GC structures and undergoing somatic hypermutation (SHM) via autophagy machinery,^28^ which, in turn, leads to increased accessibility and mutagenesis to the immunoglobulin variable region (IgV).^26^

Although the exact mechanism of lamina‐mediated mutagenesis remains unknown, current evidence suggests that disruption of Lamin B1 nuclear localization initiates chromatin relaxation and induces the formation of transcriptionally active chromatin.^14^, ^15^, ^29^, ^30^, ^31^ Among the documented, multifaceted roles of Lamin B1, previous studies have described its involvement in mediating the DNA damage response (DDR) and chromosomal instability.^32^, ^33^, ^34^, ^35^ Chromosomal translocations frequently arise from activation‐induced cytidine deaminase (AID)‐ mediated double‐strand breaks (DSBs) during B cell activation, which are driving factors in lymphomagenesis.^36^, ^37^ Under physiological conditions, B cell maturation and antibody affinity are dependent on efficient SHM and class‐switch recombination (CSR),^38^ where targeted DNA damage during CSR is resolved by the non‐homologous end joining (NHEJ) repair pathway.^39^, ^40^ An upstream mediator of NHEJ is p53‐binding protein (53BP1), which, in association with other complexes, protects double‐stranded ends and prevents the deployment of alternative repair pathways, such as homologous recombination (HR).^41^ Lamin B1 overexpression prevents the recruitment of 53BP1 to sites of DNA damage, resulting in unresolved DSBs due to inefficient NHEJ.^42^ However, while these in vitro findings shed light on Lamin B1's involvement in the DDR, its precise role in genomic instability (GI) in B cells in vivo remains unclear. Bloehdorn et al.^43^ stratified the CLL cohort of patients into GI (“genomically instable, non‐inflammatory”) and (I)EMT‐L (“epithelial‐mesenchymal‐transition‐like with inflammatory features”) subtypes. GI displays AID/APOBEC‐associated mutational signatures, consistent with a GC–experienced origin of IGHV‐mutated GI CLL. GI cases enriched for IGHV‐mutated disease harbor more alterations in genome maintenance/DNA damage–response (DDR) genes than (I)EMT‐L, which shows few such mutations. This supports a selective vulnerability in the context of AID/APOBEC activation and an insufficient mismatch repair (MMR) pathway. Given that GI CLL exhibits AID/APOBEC‐associated mutational signatures and that Lamin B1 plays a central role in GC biology and genome protection, we investigated GC‐derived lymphomas as a relevant functional model.

For that, we explored the effect of Lamin B1 depletion on GI using a hypomorphic Lamin B1 in vivo murine model in physiological GC B cells and GC‐derived lymphoma and CLL models. Complementing the functional in vitro and in vivo studies, we also implemented a multi‐omics approach that enabled us to understand how the downregulation of Lamin B1 in B cells affects the accumulation of DSBs. Our data provide novel insights into the unique role of Lamin B1 as a buffer against GI in both normal and malignant B cells. Finally, clinical analyses revealed that Lamin B1 expression is associated with survival outcomes in patients with DLBCL.

## RESULTS

### Decreased Lamin B1 is associated with GI and mutagenesis in leukemia

In analyses of the randomized CLL‐8 cohort (NCT00281918), we previously showed that low *LMNB1* expression is associated with significantly shorter progression‐free survival (PFS) and overall survival (OS) in CLL.^26^ However, the biological basis for this adverse prognostic association remains unclear. In particular, whether *LMNB1* reduction promotes GI by allowing the incorporation of DSBs has not been systematically addressed in CLL and lymphoma.

To assess whether *LMNB1* expression is associated with DNA damage‐related programs in a human disease context, we first analyzed the CLL cohort and correlated *LMNB1* expression with GI in CLL patients.^43^ We compared *LMNB1* expression among patient subgroups identified by consensus clustering and corresponding genomic categories.

**Figure 1:**
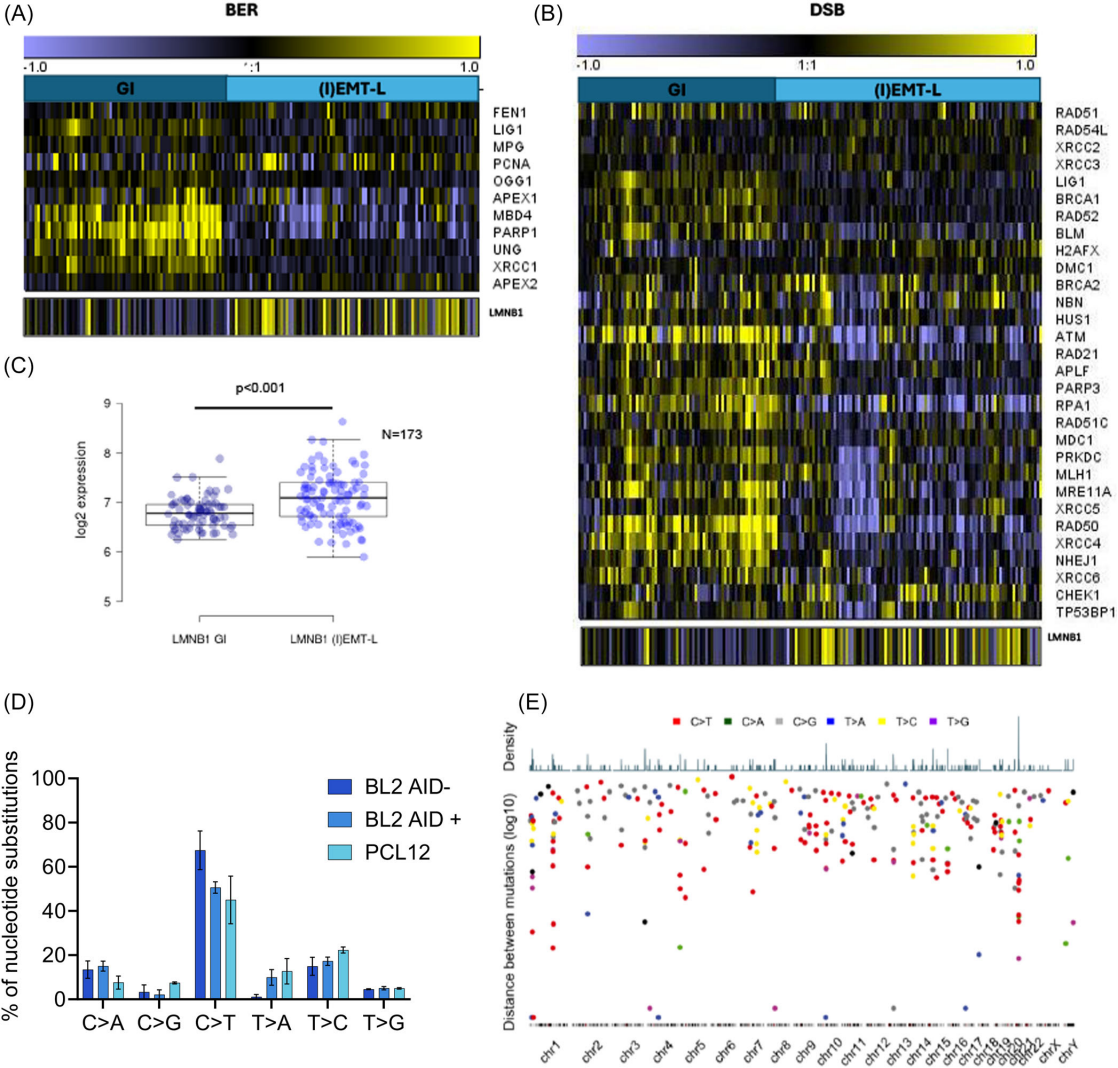
**Decreased** ***LMNB1*** **expression is associated with genomic instability (GI) in CLL and mutagenesis in malignant B cells**. **(A**, **B)** Heatmaps showing GEP for *LMNB1* and **(A)** base excision repair (BER) and **(B)** DNA DSB repair genes in GI and (I)EMT‐L CLL subtypes (*n* = 173). **(C)** *LMNB1* expression is significantly decreased in GI versus (I)EMT‐L CLL patients. **(D)** Unique somatic variants detected in siLMNB1 samples are represented as the proportion of cytosine to thymine (C>T) nucleotide substitutions. WES was performed on two independent replicates. Error bars represent ±sem. **(E)**. Inter‐mutation distance distribution of siLMNB1‐specific SNVs in BL2 AID^wt^, BL2 AID^−/−^, and PCL12 cells, identified relative to matched control conditions. The distribution of mutations was plotted in R using the karyoplotR (1.22.0) package, and hg38 was used for chromosome visualization. CLL, chronic lymphocytic leukemia.

We observed a significant decrease in *LMNB1* expression, accompanied by elevated levels of DNA repair genes (base excision repair [BER], mismatch repair [MMR]), particularly a subset of NHEJ‐related genes (*NHEJ1, RAD50, XRCC4, XRCC5*) in the GI classified CLL patients compared to (I)‐EMT‐L subtype (Figure 1A–C; Supporting Information S1: Figure S1A).

To identify the Lamin B1‐controlled genomic regions, we next performed deep whole‐exome sequencing (WES) after depleting *LMNB1* in BL2 *AID* ^*wt*^, BL2 *AID* ^−/−^, and PCL12 cell lines (Supporting Information S1: Figure S1B). We called single‐nucleotide variants (SNVs) in LMNB1‐depleted cells relative to matched controls. The resulting SNV spectra showed a predominance of cytosine‐to‐thymine (C>T) substitutions, a pattern often observed in association with AID‐related mutational processes in B cells, and this pattern was detectable across conditions irrespective of AID status^44^ (Figure 1D; Supporting Information S1: Figure S1C). To determine whether SNVs identified in Lamin B1‐depleted samples form mutational clusters, we applied the kataegis detection algorithm (katdetectr) to analyze their distribution of mutational patterns. We identified two putative kataegis foci that met less stringent criteria (≥3 variants with mean IMD ≤ 50 kb), while no kataegis foci were detected using the classical selection criteria (≥6 variants with mean IMD ≤ 1 kb). In this context, given the small number of SNVs present, detection of true, biologically significant kataegis events is unlikely. Instead, these clusters likely represent minor localized hypermutation rather than extensive APOBEC‐driven processes typical of high‐burden cancers.^45^

These findings show that decreased Lamin B1 expression is associated with GI in patients with CLL and increased mutational load in B‐cell‐derived lymphoma cells.

### Loss of Lamin B1 contributes to elevated DNA damage and recruitment of DNA damage repair proteins

The observed association between Lamin B1 and GI in CLL samples prompted us to investigate the effects of Lamin B1 reduction on GI and the recruitment of DNA repair proteins in GC‐B cell‐derived lymphoma cell lines and in vivo during B cell development in GCs.

**Figure 2:**
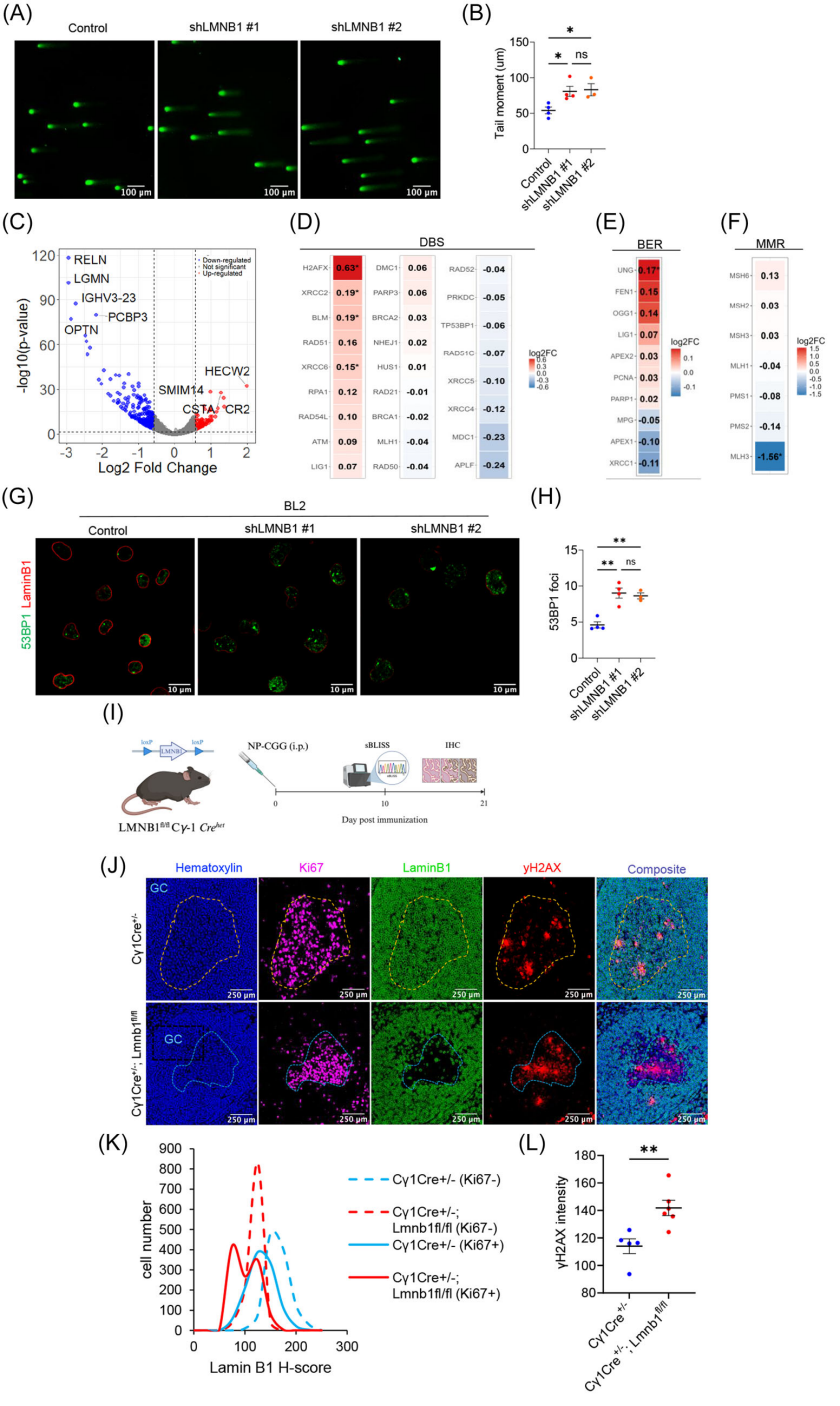
**Lamin B1 reduction translates into increased GI**. **(A)** Representative COMET assay images of BL2 control cells and stable shRNA Lamin B1 sequences. **(B)** Quantification of COMET tail length. The data represent three independent experiments. **(C)** Volcano plot comparing gene expression in shLMNB1 versus control BL2 cells. Significantly upregulated genes (padj < 0.05, log₂FC > 0.5) are shown in red; significantly downregulated genes (padj < 0.05, log₂FC < −0.5) in blue. **(D**–**F)** Log₂ fold change (shLMNB1 vs. control) of genes involved in: BER **(D)**, DSB (**E**), MMR (**F**), as introduced in Figure 1A,B and Supporting Information S1: Figure 1A. * means P < 0.05 **(G)** Immunofluorescence images showing Lamin B1 (red) and 53BP1 (green) staining in control and shLMNB1 BL2 cells. **(H)** 53BP1 dots quantification from panel g. *n* = 3 independent experiments. ** means P < 0.01. (**I**) Schematic of the conditional Lamin B1 knockout mouse model in GC B cells and associated analyses. **(J)** Representative images of multiplex immunohistochemistry for Ki67+, Lamin B1, and γH2AX markers. **(K)** Lamin B1 histogram showing reduction in GCs (Ki67+ structures) upon control (*Cγ1Cre^+/−^*) and Lamin B1‐floxed (*Cγ1Cre* ^+/−^; *Lmnb1* ^*fl/fl*^) mice in Ki67− (non‐GC) and Ki67⁺ (GC) regions. **(L)** Quantification of γH2AX intensity in GC regions of control versus Lamin B1 knockout mice. GI, genomic instability.

We generated doxycycline‐inducible Lamin B1 knockdown in GC‐derived lymphoma cell lines. Transduced BL2 (Burkitt's lymphoma) and OCI‐LY8 (GCB DLBCL) cells were analyzed for GFP positivity by flow cytometry upon addition of doxycycline (DOX) (Supporting Information S1: Figure S2A,B), and the efficacy of short‐hairpin RNA (shRNA)‐mediated Lamin B1 knockdown was assessed by western blot analysis (Supporting Information S1: Figure S2C). Alkaline single‐cell electrophoresis (comet) assay showed higher tail moments in Lamin B1‐depleted cells, revealing increased DNA damage after Lamin B1 knockdown in the BL2 cell line (Figure 2A,B).

GI is a driver of transcriptional change,^46^ and persistent DNA damage signaling and altered chromatin organization can reprogram gene expression. Previous studies have demonstrated that Lamin B1 contributes to gene repression within LADs and that its removal modulates the transcriptional profile in various cancer models.^9^, ^20^, ^31^ To gain insight into the transcriptional landscape modulated by Lamin B1, we performed RNA‐seq analysis of shLMNB1‐treated BL2 cells and controls. Using the DESeq2 algorithm, we detected differentially expressed genes in BL2 cells upon Lamin B1 silencing; 526 genes were significantly dysregulated (padj < 0.05, Log2FC ± >0.5) (Figure 2C; Supporting Information S1: Table S1). We next examined changes in gene expression within the BER, DSB, and MMR pathways previously assessed in CLL patients. We compared shLMNB1 and control conditions using log_2_ fold change (log_2_FC).

RNA‐seq revealed an overall tendency toward increased expression of several DNA‐repair transcripts in shLMNB1 cells; however, only a subset remained statistically significant after multiple‐testing correction (DESeq2; BH‐adjusted padj < 0.05) (Figure 2D–F and Supporting Information S1: Table S2). In addition, many of these genes encode proteins whose functional activation is primarily regulated posttranslationally (e.g., phosphorylation), which may not be reflected at the mRNA level.^47^

As an additional control, we assessed Lamin B1 protein levels before performing the RNA‐seq experiment (Supporting Information S1: Figure S2D) and further validated the expression of LMNA, LMNB1, LMNB2, and APOBEC family genes in our RNA‐seq dataset (Supporting Information S1: Figure S2E,F). The results showed that the relative expression of all queried genes remained unchanged in shLMNB1 compared with control BL2 cells, except for Lamin B1, whose expression was significantly reduced under the knockdown condition. These findings indicate that the changes observed in our system are specific to Lamin B1 depletion.

To validate the upregulation of key proteins in the DSB pathway, we performed immunofluorescence (IF) and western blot analyses of γ‐H2AX and 53BP1. A marked increase in 53BP1 foci per cell was observed following Lamin B1 depletion (Figure 2G,H and Supporting Information S1: Figure S2G), indicating an enhanced DNA damage response in BL2 and OCI‐LY8 cell lines. Similarly, γ‐H2AX (Supporting Information S1: Figure S2H) levels were elevated in OCI‐LY8 Lamin B1 knockout cells, further supporting the activation of DNA repair pathways. Additionally, to assess that Lamin B1 knockdown does not prevent DNA repair proteins from being recruited to DNA damage sites, we co‐stained control and shLMNB1 cells with 53BP1 and γ‐H2AX. First, we confirmed Lamin B1 reduction upon Lamin B1 knockdown in the cells prepared for IF (Supporting Information S1: Figure S2I). Representative images in Supporting Information S1: Figure S2J show increased γ‐H2AX puncta in shLMNB1 cells, consistent with Supporting Information S1: Figure S2H. A fraction of γ‐H2AX foci co‐localize with 53BP1, indicating that Lamin B1 depletion does not prevent repair factor recruitment (Supporting Information S1: Figure S2K). Instead, Lamin B1 loss increases the overall lesion burden and the fraction of γ‐H2AX‐positive foci that are 53BP1‐negative relative to controls.

Next, we generated a GC B cell‐specific inducible *LMNB1* knockout mouse model to evaluate Lamin B1's impact on DSBs under physiological conditions (Figure 2I). Using multiplex immunohistochemistry (mIHC) (Figure 2J), we identified GCs histologically using the Ki67 marker and then evaluated the levels of Lamin B1 (Figure 2K) and γ‐H2AX (Figure 2L). Our observations showed that the depletion of Lamin B1 alone is sufficient to increase the γ‐H2AX signal. Additionally, we isolated PNA+ (GC B) and PNA‐ (non‐GC B) cells (Supporting Information S1: Figure S2L) and, by IF, confirmed that γ‐H2AX was increased in Lamin B1 KO PNA+ cells, supporting our IHC findings.

Altogether, these results highlight the crucial role of Lamin B1 in maintaining genomic integrity and that its loss alone increases DNA DSBs.

### Genome‐wide profiling of DSB topology in human and mouse Lamin B1‐depleted B cells

By modulating chromatin accessibility, Lamin B1 helps orchestrate GC B cell programs and preserves GC architecture.^28^ Alterations in genome accessibility and transcriptional programs often manifest as phenotypic changes at the tissue level. To determine whether these molecular alterations were reflected in GC organization, we examined spleen architecture by immunohistochemical staining for BCL6, which delineates GCs (Supporting Information S1: Figure S3A). We observed that *Cγ1Cre* ^+/−^ *;Lmnb1* ^*fl/fl*^ mice displayed a higher number of GCs compared with *Cγ1Cre* ^+/−^ control (Supporting Information S1: Figure S3B), although the average GC size, measured by area, was significantly reduced (Supporting Information S1: Figure S3C).

**Figure 3:**
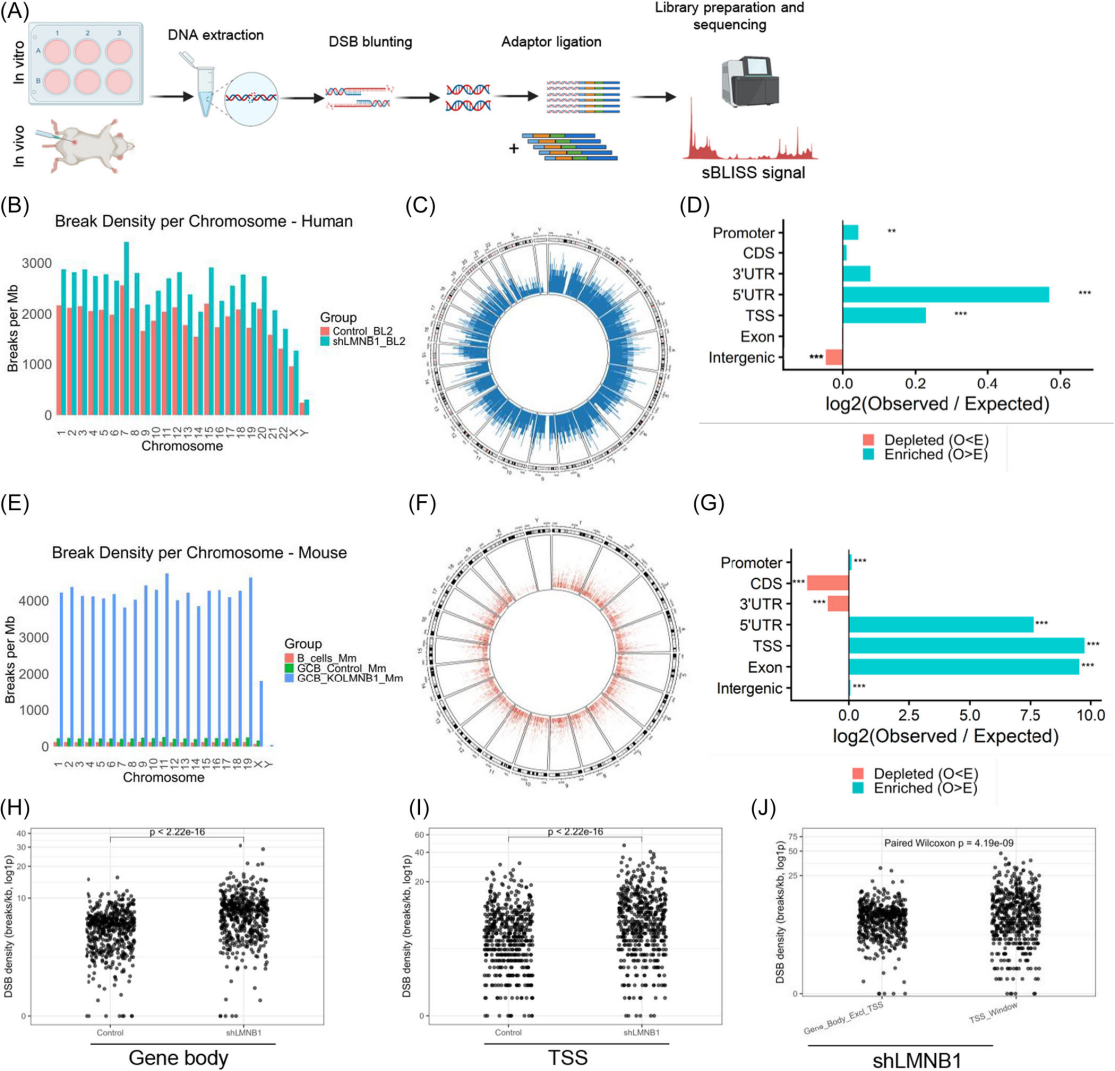
**Lamin B1 protects the genome from high‐density DNA breaks**. **(A)** Schematic diagram illustrating the experimental workflow for generating sBLISS data. **(B)** Bar plot showing the distribution of DSB density (breaks per megabase) across chromosomes in BL2 cells under control and shLMNB1 conditions. **(C)** Circoplot showing the unique hotspot distribution in shLMNB1 compared to the control in BL2 cells. **(D)** Barplot showing increase/decrease in hotspots in shLMNB1 compared to control in seven genomic features. Barplot represents the log2 observed/expected (log2 O/E) number of hotspots per category. (FDR/q‐values) are obtained after P‐value adjusted using Benjamini‐Hochberg (BH) (*FDR < 0.05; **FDR < 0.01; ***FDR < 0.001). **(E)** Bar plot showing DSB density (breaks per megabase) across chromosomes in ex vivo samples: nongerminal center Naïve B cells (B_cells_Mm), *Cγ1Cre* ^+/−^ GC B cells (GCB_Control_Mm), and *Cγ1Cre* ^+/−^ *; Lmnb1* ^*fl/fl*^ GC B cells (GCB_KOLMNB1_Mm). (**F**) Unique DSB hotspots distribution in *Cγ1Cre* ^+/−^ *;* Lmnb1^fl/fl^ compared to *Cγ1Cre* ^+/−^ *; Lmnb1* ^*fl/fl*^ GC B cells. **(G)** Barplot showing increase/decrease in hotspots in shLMNB1 compared to control in seven genomic features. Barplot represents the log2 observed/expected (log2 O/E) number of hotspots per category. (FDR/*q*‐values) are obtained after P‐value adjusted using Benjamini‐Hochberg (BH) (*FDR < 0.05; **FDR < 0.01; ***FDR < 0.001). **(H)** Dotplot showing DSB/Kb in DEGs body regions comparing control and shLMNB1. **(I)** Dotplot showing DSB/Kb in DEGs TSSs comparing control and shLMNB1. **(J)** Dotplot comparing DSB/Kb in the gene body and TSS in the shLMNB1 condition using DEGs from RNAseq.

Given the observed architectural and transcriptional disruptions, we next sought to determine whether these changes were accompanied by increased GI. We performed genome‐wide mapping of DNA DSBs to identify which Lamin B1‐protected regions were preferentially prone to breakage, revealing structural vulnerabilities. For this, we employed breaks labeling in situ and sequencing (BLISS) (Figure 3A and Supporting Information S1: Figure S4A,B) to map regions of increased DNA fragility.^48^

The BL2 cell line, mouse splenic GC B cells (B220⁺, CD95⁺, GL7⁺), and naïve B cells (B220⁺, CD95⁻, GL7⁻) were used for the analysis. Principal Component Analysis (PCA) revealed replicate consistency and sample variability between conditions in in vivo and in vitro scenarios (Supporting Information S1: Figure S4C,D).

We first quantified DSBs per megabase (Mb) for each chromosome. Our in vitro BL2 Lamin B1 knockout model showed increased DSBs across all chromosomes (Figure 3B). Regional analysis revealed that Lamin B1 deficiency led to a broader increase in DSB burden, particularly at 5′ and 3′‐UTR, TSS, and exons (Supporting Information S1: Figure S4E). When analyzing DSB hotspots, we observed that Lamin B1 depletion in vitro led to a markedly higher number of unique genome‐wide DSB hotspots compared with control cells (Figure 3C). Complementary to this, the distribution of shLMNB1 hotspots against the genomic background (control BL2) in vitro appeared nonrandomly distributed across genomic features (Figure 3D). Our log_2_(observed/expected) analysis shows that the strongest significant enrichment is in gene‐regulatory regions proximal to transcription start sites (TSS, 5′‐UTR, and promoter intervals). In contrast, intergenic regions were depleted for hotspots relative to control cells (Figure 3D).

Consistent with our findings in the human BL2 cell line, *LMNB1* depletion in mouse GC B cells increased DSB burden across the genome. Compared with control GC B cells (*Cγ1Cre* ^+/−^), *Cγ1Cre* ^+/−^; *Lmnb1* ^*fl/fl*^ GC B cells showed a marked increase in DSB density per chromosome and across annotated genomic regions (Figure 3E and Supporting Information S1: Figure S4F), together with a higher number of unique DSB hotspots (Figure 3F). In addition to the global increase in DSB and hotspots, Lamin B1 ablation translated into a significant redistribution of hotspots in TSS, 5′‐UTR, and promoter‐proximal regulatory regions compared to the genomic background (*Cy1Cre* ^*+/−*^) (Figure 3G). This indicates that the increase in break sites is not distributed uniformly across all genomic categories but is biased toward regulatory architecture near gene starts.

Upon LMNB1 loss, hotspot signal values rise in both the BL2 cell line and mouse GC B cells. The genomic distribution of hotspots, together with the dynamic range of their break frequencies, is largely conserved in human and mouse samples (Supporting Information S1: Figure S4G). Together, the human and mouse analyses support a conserved role for Lamin B1 in safeguarding genome integrity at core transcriptional regulatory regions, revealing a shared breakage‐prone landscape that becomes unmasked when Lamin B1 function is disrupted.

We next leveraged our BL2 RNA‐seq data to assess DSB distribution across gene bodies and TSSs of DEGs. Upon *LMNB1* reduction, DSB accumulation increased at both TSSs and gene bodies of DEGs (Figure 3H,I). Furthermore, within the *LMNB1* knockdown condition, TSSs displayed a significantly higher DSB density per kilobase than gene bodies (Figure 3J), indicating that DSB formation is not random and is preferentially enriched at promoter‐proximal regions, consistent with the sBLISS findings shown in Figure 3D.

Finally, we asked whether genes commonly mutated in DLBCL^49^ and driver genes in CLL^50^ coincide with DSBs and hotspots revealed in our system. Of the 142 DLBCL genes commonly mutated in this disease, 134 (≈94%) overlapped with at least one DSB within the gene body in the shLMNB1 condition. Similarly, we found that 96/97 (98.96%) CLL driver genes overlapped with at least one DSB in shLMNB1 cells. We then asked whether driver genes in DLBCL and CLL also harbored shLMNB1‐specific DSB hotspots. We found that 76/142 (53.5%) in DLBCL and 66/97 (68%) in CLL contained hotspots unique to the shLMNB1 condition (Supporting Information S1: Tables S3 and S4). These results support a model in which *LMNB1* downregulation is associated with recurrent accumulation of DNA breaks in cancer driver genes. Moreover, this data suggests a mechanism not restricted to a single disease context, and that may contribute to GI in both CLL and GC‐derived lymphomas. These data also indicate that Lamin B1 safeguards genome stability in GC B cells by limiting the formation of nonrandom, promoter‐proximal DNA DSBs, acting as a key structural determinant of genome integrity in B cells.

### Lamin B1 loss enhances DNA fragility at LADs and rewires functional gene networks

**Figure 4:**
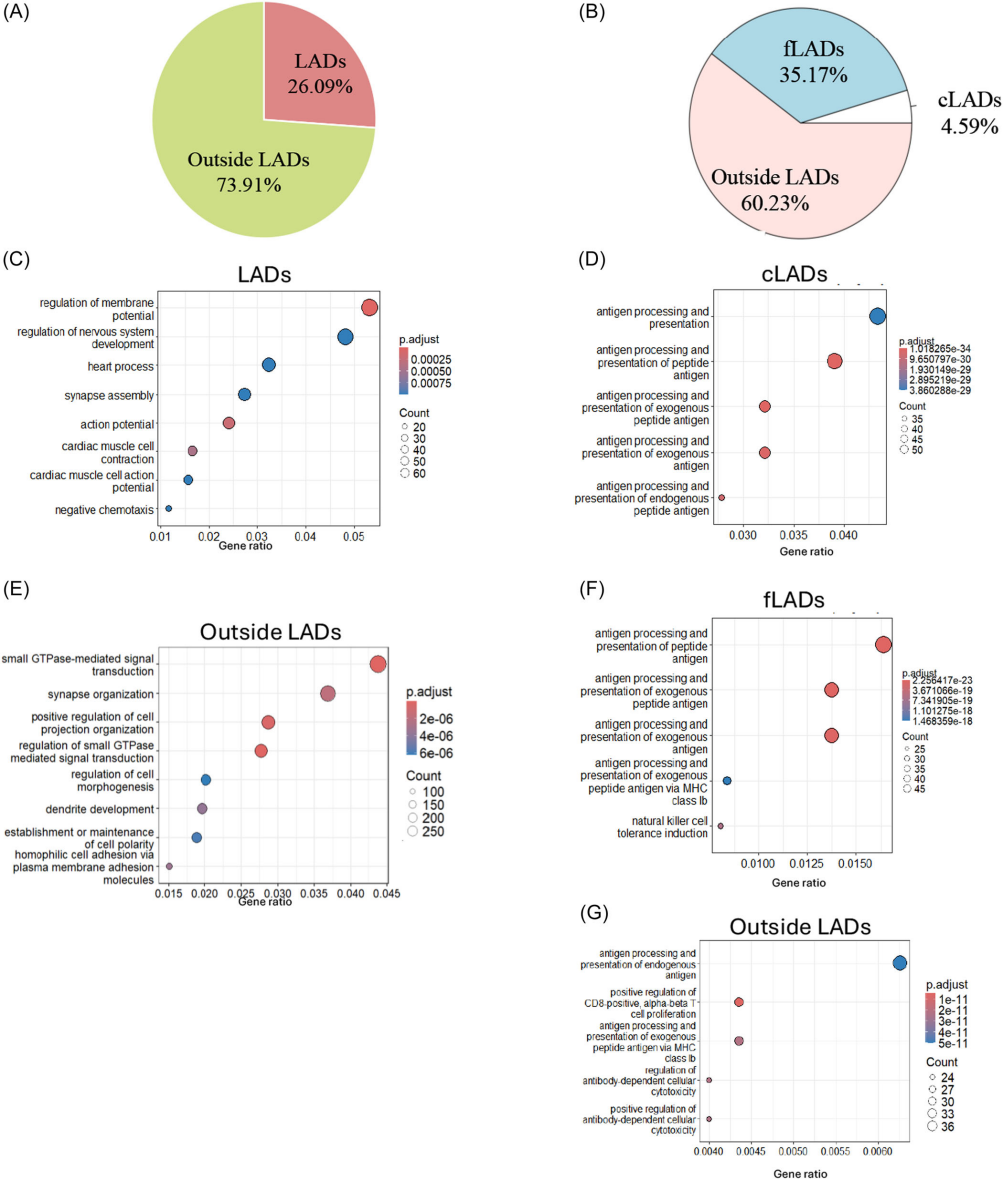
**Lamin B1 downregulation impacts genome architecture, rewiring the functional genomic landscape of the cell**. **(A)** Pie chart showing the genomic distribution of unique Lamin B1‐dependent DSB hotspots classified into LADs and outside‐LAD regions in the BL2 cell line. **(B)** Pie chart showing the genomic distribution of unique Lamin B1‐dependent DSB hotspots classified into LADs (cLADs and fLADs) and outside‐LAD regions in *Cγ1Cre* ^+/−^; *Lmnb1* ^*fl/fl*^ GC B cells. Gene Ontology (GO) Biological Process (BP) enrichment of genes associated with LADs **(C)** and outside‐LADs **(D)** from A **(E–G)**. Dotplot showing significantly enriched GO terms from genes associated with cLAD **(E)**, fLADs **(F)**, and outside‐LADs **(G)** contexts from B. DSB, double‐strand breaks; LAD, lamina‐associated domains.

We next sought to investigate how the reduction of Lamin B1 impacts the functional genomic landscape in lymphoma and in GC B cells. We utilized the sBLISS data to examine the genomic distribution of unique DSB hotspots in Lamin B1‐depleted cells, both in vitro (Figure 4A) and in vivo (Figure 4B), by dichotomizing the genome into LAD and non‐LAD regions.

In in vitro settings, we found that 26.09% of hotspots occurred within LADs. In comparison, 73.91% were localized outside LADs. In the mouse settings, the distribution of hotspots in GC B cells exhibited a similar trend, finding 39.76% of hotspots located within LADs. These data indicate that Lamin B1 depletion causes widespread disruptions to genome architecture that extend beyond LADs, altering overall nuclear and chromatin organization.

We next asked whether hotspot‐dense genes are biased toward specific biological programs, suggesting that particular gene networks are disproportionately vulnerable to *LMNB1* perturbation‐associated GI. To address this, we performed in silico Gene Ontology (GO) enrichment analysis on genes harboring >1 DSB hotspot, stratifying genes by LAD‐associated versus non‐LAD localization in human BL2 and mouse GC B cells.

In human BL2 cells, LAD‐associated hotspot‐dense genes were enriched for GO terms dominated by electrical excitability and tissue physiology, consistent with hotspot clustering within LADs, preferentially impacting genes linked to membrane potential and specialized physiological functions (Figure 4C). In contrast, hotspot‐dense genes outside LADs were enriched for pathways related to cellular signaling and structural remodeling, including processes governing cytoskeletal organization, cell polarity, and cell–cell interactions (Figure 4D). In mouse GC B cells, the functional landscape shows hotspot‐associated genes within LADs (cLADs and fLADs) were enriched for immune‐associated functions, including antigen processing and presentation (Figure 4E,F). Similarly, hotspot‐associated genes outside LADs were enriched for immune regulatory pathways, proliferation, and cytotoxicity (Figure 4G). Together, these data suggest that, in mice, GI outside lamina‐associated chromatin preferentially maps to immune regulatory networks.

Finally, to identify conserved responses, we compared enriched terms across species and found a shared set of pathways associated with hotspot accumulation in both human and mouse, including processes linked to actin cytoskeleton‐dependent migration, DNA replication, and nuclear division (Supporting Information S1: Table S5). The presence of hotspots within these genes does not necessarily imply pathway dysregulation; rather, it suggests that these gene programs may be more vulnerable and therefore more likely to be affected under LMNB1 perturbation.

### Lamin B1 loss promotes DSBs at AICDA and novel intergenic motifs

**Figure 5:**
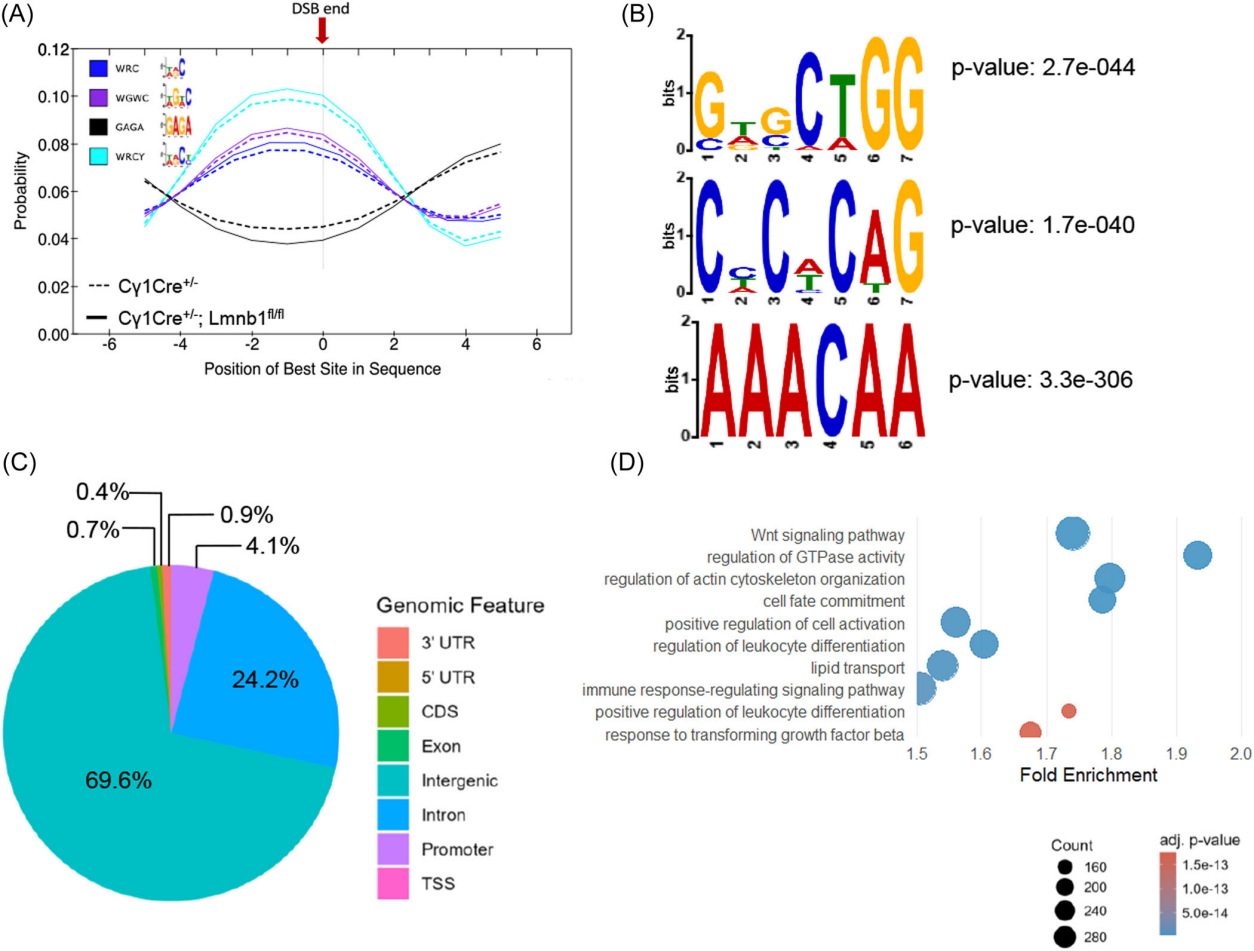
**Lamin B1 loss promotes DSBs at AICDA motifs and defines de novo nucleotide patterns**. **(A)** AID hotspots (WRC, WRCY, and WGCW) location probability associated with DSBs in *Cγ1Cre* ^+/−^; *Lmnb1* ^*fl/fl*^ compared to *Cγ1cre* ^+/−^ GC B cells. **(B)** Top 3 enriched de novo motifs in *Cγ1cre* ^+/−^; *Lmnb1* ^*fl/fl*^ GC B cells. **(C)** Pie chart showing percentage of de novo motifs found at different genomic regions in *Cγ1cre* ^+/−^; *Lmnb1* ^*fl/fl*^. **(D)** GO enrichment results showing deregulated biological processes associated with genes annotated near motif locations (±3 kb of a TSS) derived from the discovered motif sites. DSB, double‐strand breaks; GO, Gene Ontology.

GC B cells are the origin of many lymphomas and rely on AID as the primary mutator enzyme.^51^ We therefore investigated how Lamin B1 depletion affects DNA damage at AID targets and newly de novo motifs. We first focused on AID hotspots (WRC, WRCY, and WGCW)^52^ using DSB hotspots from the *Cγ1Cre* ^+/−^ and the *Cγ1Cre* ^+/−^; *LMNB1* ^*fl/fl*^ mouse models. Using the CentriMo algorithm, we determined the probability of motif locations within the DNA sequence associated with DSBs (Figure 5A and Supporting Information S1: Figure S5A). As expected, the AID target motif sequence was located near the center of the sequence in the *Cγ1Cre* ^+/−^ and the *Cγ1Cre* ^+/−^; *Lmnb* ^*fl/fl*^, corresponding to the original DSB. A non‐AID motif sequence (GAGA) was not located at the center of the DSB sequence, confirming the specificity of the AID‐induced DSB site in GC B cells.

Using MEME Suite (v5.5.8), we identified several de novo motifs significantly enriched around Lamin B1‐associated DSB hotspots (Figure 5B and Supporting Information S1: Figure S5B). The top motifs included GWGCTGG (*E* = 1.7e−40), found at 2710 sites, and CHCWCAG (*E* = 1.7e−40), a GC‐rich motif frequently observed in enhancer‐like regions. Additionally, AAACAA and TTTCTT, which are AT‐rich motifs that may reflect fragile genomic regions or regulatory repressor elements. Notably, the GWGCTGG motif contains a conserved central CTGG core and, based on Tomtom similarity searches against JASPAR 2024 CORE and UniPROBE, most closely matched motifs associated with the hematopoietic TFs SPI1 (PU.1) and MEF2C. These TFs have prominent roles in hematopoiesis, regulating lineage specification and stem cell maintenance.^53^, ^54^, ^55^, ^56^ When dysregulated, these have been linked to malignant transformation in acute myeloid leukemia (AML) and high‐risk B‐cell acute lymphoblastic leukemia (B‐ALL).^57^, ^58^, ^59^

To determine their genomic distribution, we mapped the de novo motifs and found that most reside in intergenic (69.6%), intronic (24.2%), and promoter (4.1%) sequences (Figure 5C). This pattern indicates that DSBs arising after Lamin B1 depletion occur non‐randomly and are enriched in regulatory regions, underscoring the NL's role in safeguarding genome integrity.

To further extend the analysis, the de novo motifs were then matched to known TF motifs using Tomtom (MEME Suite). To contextualize the biological relevance of matched TFs, we utilized CistromeDB, querying publicly available ChIP‐seq data across different cell types to evaluate TF binding‐site enrichment near our input regions, and then computed the GIGGLE similarity scores. High‐scoring factors, such as LEO1, MED1, KMT2B, and STAT5, emerged as candidates potentially involved in DSB‐associated regulatory processes (Supporting Information S1: Figure S5C). To understand the chromatin context of these breaks, we again applied GIGGLE enrichment analysis to reference epigenomic datasets.

Our analysis showed that DSB hotspots unique to Lamin B1‐deficient GC B cells preferentially occur in regions associated with repressive histone marks, particularly H3K9me3 and H3K27me3 (Supporting Information S1: Figure S5D). By contrast, we observed only moderate overlap with core histone H3 and enhancer‐associated H3K27ac, and minimal enrichment for transcription‐elongation marks (H3K36me3, H3K79me2) or less common histone variants (Supporting Information S1: Figure S5D). We additionally leveraged the GSE143293 dataset to assess, at the peak level, the overlap between Lamin B1‐depleted unique DSB hotspots and histone ChIP–Seq peaks generated in GC B cells. Supporting Information S1: Figure S5E shows that 4314 of 24,810 hotspots (17.39%) overlapped H3K27me3 peaks, whereas only 39 of 24,810 hotspots (0.16%) overlapped H3K27ac peaks. Thus, the unique Lamin B1‐depleted hotspots exhibit markedly greater overlap with a repressive chromatin mark than with an active enhancer mark, supporting the conclusion that these hotspots are preferentially associated with repressed chromatin states.

To identify biological processes potentially affected by motif‐associated DNA damage, we extended the genomic coordinates of de novo‐discovered motifs by ±3 kb and mapped them to the nearest TSS. This mapping yielded a set of genes near the motif hotspots. GO enrichment analysis revealed that these genes were significantly associated with pathways involved in actin cytoskeleton organization, cell fate commitment, and Wnt signaling (Figure 5D and Supporting Information S1: Table S6): processes implicated in hematological tumor progression and plasticity.^60^, ^61^, ^62^ Additionally, enrichment of immune‐related terms, such as leukocyte differentiation and immune response–regulating signaling, further suggests that DSB accumulation at motif‐associated regions can shape both structural and immunomodulatory programs in lymphoma.

### Decreased *LMNB1* expression is associated with poor clinical outcomes of DLBCL

**Figure 6:**
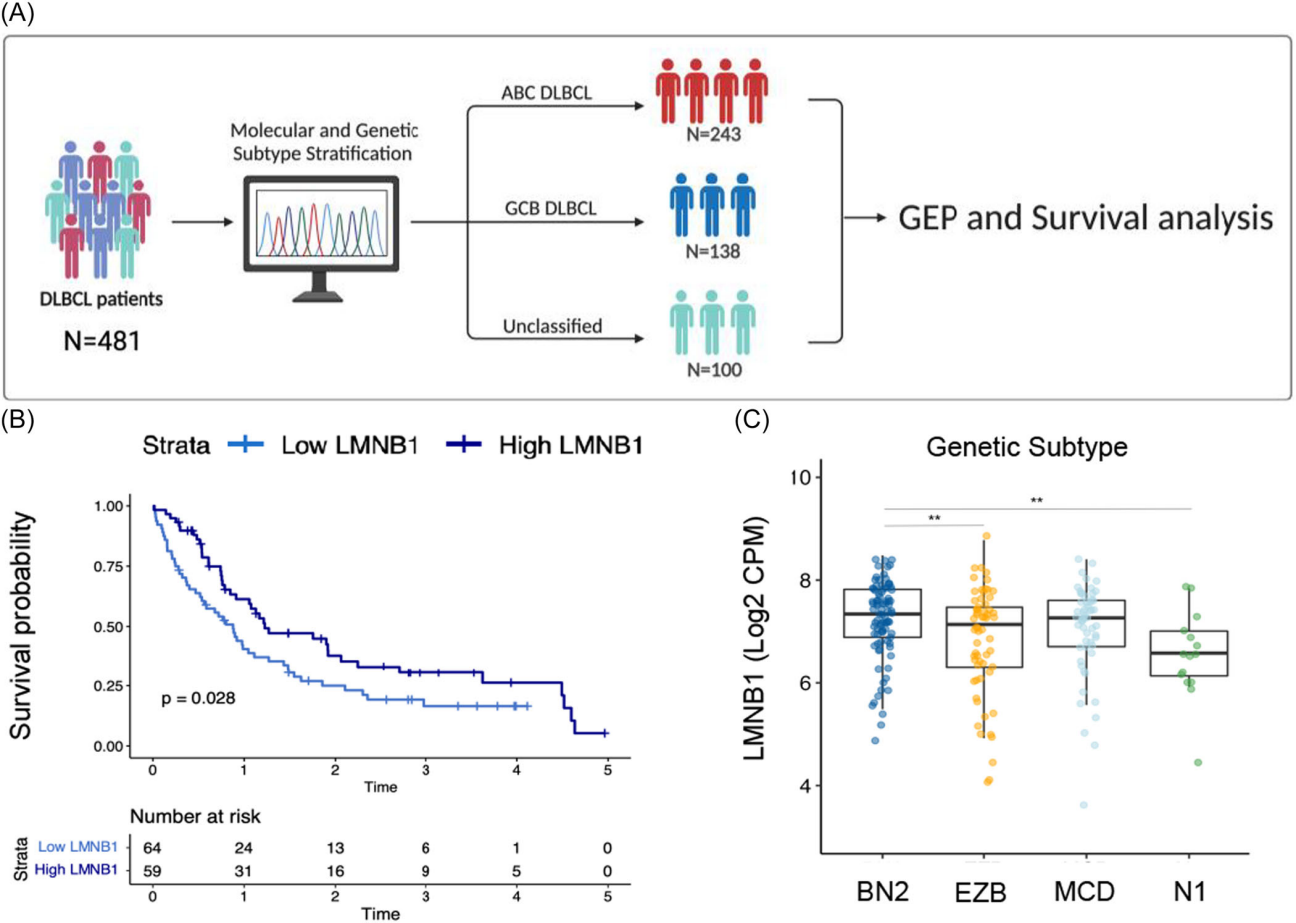
**Decreased LMNB1 expression is associated with worse clinical outcome in DLBCL**. **(A)** Workflow diagram of clinicopathological analysis in DLBCL patients. **(B)** Kaplan−Meier curve estimates of 5‐year PFS based on median LMNB1 expression in DLBCL patients (*N* = 123). **(C)** *LMNB1* expression is decreased in N1 and EZB genetic DLBCL subtypes (*N* = 220). Unpaired *t*‐test was used for the statistical analysis *P ≤ 0.05; **P ≤ 0.01; ns, not significant. DLBCL, diffuse large B‐cell lymphoma; PFS, progression‐free survival.

In hematological malignancies, including CLL and AML, reduced *LMNB1* expression is associated with adverse clinical outcomes.^9^, ^26^ Considering the profound involvement of *LMNB1* in processes implicated in hematological tumor progression and plasticity (Figure 5D), we next investigated the clinical significance of Lamin B1 in GC lymphomas, including DLBCL^63^ (Figure 6A). Remarkably, we observed a significantly shorter 5‐year survival rate in all patients with low *LMNB1* expression, as stratified by the median (Figure 6B), therefore defining the clinical relevance of *LMNB1* in DLBCL. We next investigated whether the *LMNB1*‐mediated survival difference is associated with the cell of origin and genetic subtype.

While *LMNB1* expression did not vary across stages in the ABC subtype (Supporting Information S1: Figure S6A), a stage‐associated decline was observed in GCB DLBCL, reaching its lowest levels at intermediate stages (II–III) before stabilizing at stage IV (Supporting Information S1: Figure S6B). This indicates a non‐linear, subtype‐specific association with stage rather than progressive depletion with advancing disease. In contrast, ABC‐DLBCL shows no stage‐associated differences, supporting the idea that *LMNB1* biology differs between molecular subtypes. Genetic classification revealed that *LMNB1* expression was reduced in the N1 and EZB subtypes (Figure 6C), linking Lamin B1 loss to genetically defined DLBCL subgroups.

Finally, to define the clinical impact of Lamin B1 at the protein level, we used DLBCL tissue microarrays, linked to clinical outcome data.^64^ Supporting Information S1: Figure S6C,D suggest that low Lamin B1 protein levels, as dichotomized by the mean, predicted poorer survival in patients who were event‐free beyond 24 months (Supporting Information S1: Figure S6D).^65^, ^66^ These findings suggest that Lamin B1 is not primarily a marker of early aggressive disease, but may instead reflect longer‐term disease behavior in late survivors, including disease persistence, relapse propensity, or evolutionary fitness.

## DISCUSSION

**Figure 7:**
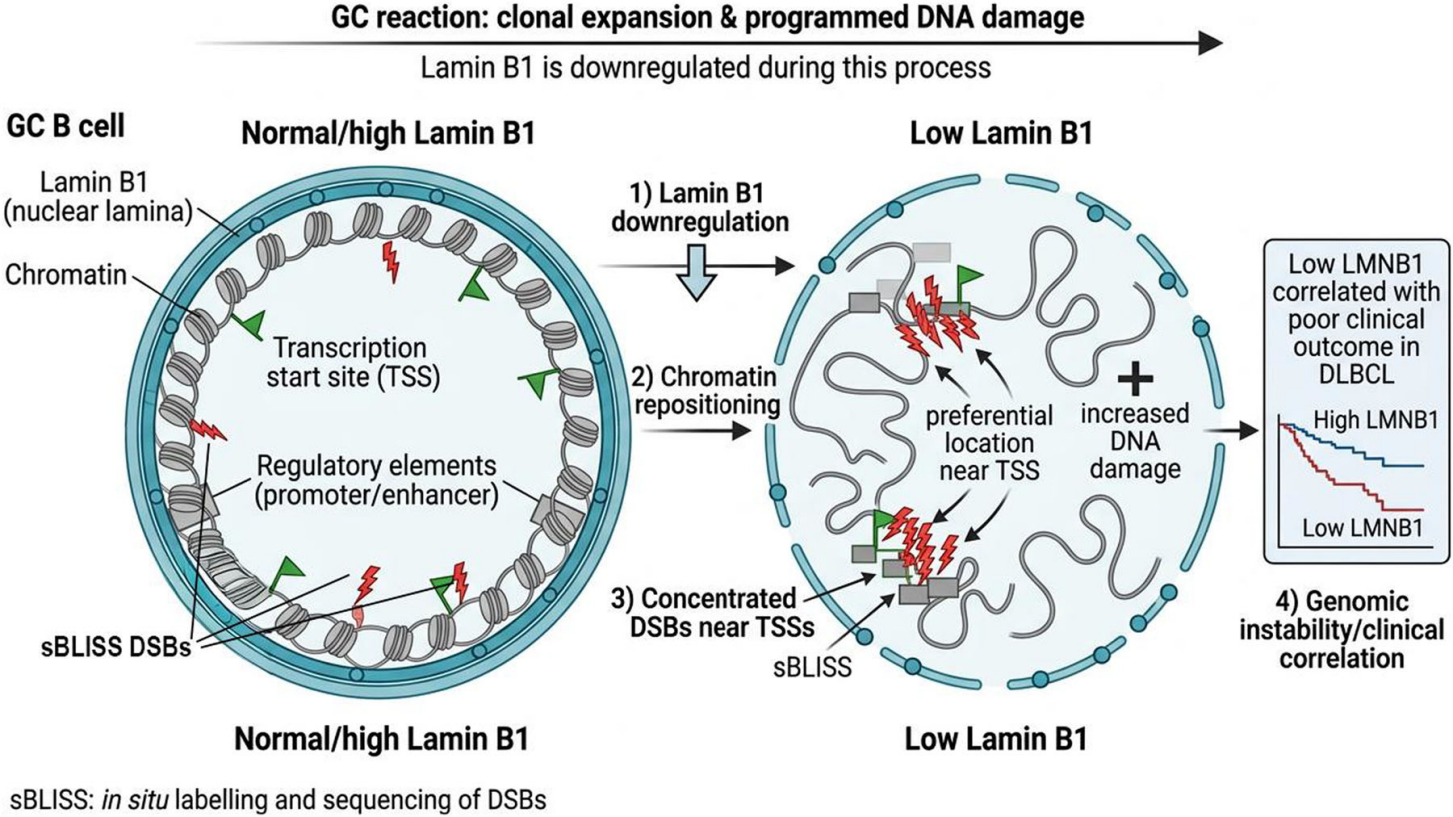
**Lamin B1 protects GC‐transitioning B cells from increased DNA damage and nonrandomly distributed DNA DSBs at regulatory elements**. During the GC reaction, GC B cells undergo rapid clonal expansion coupled with programmed DNA damage, a process accompanied by progressive Lamin B1 downregulation. Under normal or high Lamin B1 conditions, chromatin is anchored at the nuclear periphery via LADs, maintaining a spatially organized architecture in which DSB hotspots are distributed in a manner consistent with regulated transcriptional activity. Aberrant Lamin B1 reduction disrupts this organization by triggering global chromatin repositioning away from the nuclear lamina and into the nuclear interior. DSB hotspots mapped by sBLISS accumulate non‐randomly at accessible regulatory regions, suggesting that Lamin B1 loss reshapes the nuclear damage landscape in a topology‐dependent manner that selectively targets the regulatory genome rather than simply increasing global DNA damage. Consistent with these mechanistic findings, low LMNB1 expression correlates with significantly worse overall survival in DLBCL compared to high LMNB1, positioning Lamin B1 as a critical guardian of genome integrity whose loss has direct pathological consequences in B‐cell malignancy. DLBCL, diffuse large B‐cell lymphoma; DSB, double‐strand breaks; TSS, transcription start sites.

While controlled DNA damage is essential for humoral immunity, it can inadvertently generate chromosomal translocations and other genomic alterations that initiate lymphomagenesis.^67^, ^68^ The mechanisms safeguarding against such GI remain incompletely understood. Here, we investigated the role of Lamin B1 in maintaining genome stability in human lymphoma cells and normal murine GC B cells. We found that Lamin B1 depletion in B cells was associated with widespread transcriptional reprogramming, clustering of DNA DSBs, and poor survival outcomes in DLBCL (Figure 7).

Low *LMNB1* expression and GI were independently reported as adverse prognostic factors in the CLL8 and REACH CLL trial cohorts.^26^, ^43^ Our findings showed that *LMNB1* expression is particularly reduced in CLL cases of the GI subtype (REACH trial cohort), providing evidence that loss of Lamin B1 is associated with GI in CLL and possibly other B‐cell‐derived malignancies.

Upon B cell activation, reduced Lamin B1 nuclear incorporation was shown to be accompanied by a higher mutational load within the *IghV* cluster.^26^ In addition, mutational clustering of primarily C to T substitutions, consistent with the AID mutagenic signature, occurs outside the IghV region in Lamin B1‐depleted lymphoma cells. However, similar mutational patterns in BL2 *AID* ^*wt*^ and BL2 *AID* ^*−/−*^ cells suggest the potential involvement of other APOBEC deaminases, such as APOBEC3C and APOBEC3G, which are also known to drive mutagenesis in myeloma.^69^

Lamin B1 depletion from the nuclear periphery is associated with changes in chromatin organization, DNA accessibility, and damage dynamics.^15^, ^30^, ^70^, ^71^ Here, we also show that increased accessibility in Lamin B1‐deficient cells drives a targeted, AID‐independent increase in mutations outside Ig loci, as evidenced by the non‐uniform distribution of SNV clusters. To our knowledge, this is the first demonstration that reduced *LMNB1* expression elicits spontaneous DNA damage in B cells in vitro and in vivo.

The increased localization of 53BP1 clusters in response to Lamin B1 depletion is also consistent with reports that Lamin B1 controls the recruitment of 53BP1 to the damaged site in SV40‐transformed human fibroblasts and U2OS cells.^42^ In that study, *LMNB1* overexpression impaired 53BP1 accumulation at sites of DNA damage. In our system, *LMNB1* downregulation is associated with increased DNA accessibility and a higher DSB burden (Figures 2 and 3), and we correspondingly observe robust recruitment of 53BP1 to DNA‐damaged sites, indicating that DNA damage signaling and repair factor engagement remain active under *LMNB1* loss. Interestingly, the NHEJ pathway is a primary repair mechanism in B cells and is biased toward DSBs located within relaxed chromatin.^72^ Our data suggest that the fast, error‐prone NHEJ pathway is involved in DSB repair, as indicated by increased 53BP1 colocalization with γ‐H2AX in Lamin B1‐depleted cells, together with higher overall levels of both 53BP1 and γ‐H2AX compared with control cells.

Lamin B1 is essential for mouse development and cell differentiation, as established in several in vivo models.^20^, ^73^, ^74^ To investigate its role in GC biology while avoiding the deleterious effects of global *LMNB1* deletion, we generated a conditional GC B cell‐specific *LMNB1* knockout mouse model.

Our mouse model demonstrates that Lamin B1 reduction alone is sufficient to increase DNA damage in GC B cells and induce a widespread increase in DNA DSBs. Furthermore, genome‐wide analysis of hotspot landscape in in vitro and in vivo models revealed that DSB hotspots accumulated predominantly within DNA regulatory regions and TSS. This is particularly meaningful because it provides, to our knowledge, the first evidence that Lamin B1 preferentially associates with specific genomic regions rather than being uniformly distributed across the genome.

Translating these findings to the clinical setting, integration with a high‐resolution human DSB map showed that 94% (134/142) of the most frequently mutated genes in DLBCL, and 99% of the corresponding genes in CLL, contained at least one DSB. Moreover, a substantial fraction of these genes harbored DSB hotspots unique to the *LMNB1*‐depleted condition, 54% in DLBCL, and 69% in CLL. Together, these patterns suggest a protective functional role for Lamin B1 at key driver loci and indicate that this mechanism is not restricted to a single disease context but may contribute more broadly to GI in both CLL and DLBCL.

While Lamin B1 is known to regulate genome positioning in both LADs and non‐LADs,^15^, ^75^ our data show that its loss increases DSBs in non‐LAD regions in vivo and in vitro, highlighting its role as a broader chromatin protector beyond the classical LADs.

We found that DSBs were preferentially enriched around TSSs in Lamin B1‐deficient cells. This distribution aligns with the well‐characterized off‐target activity of AID outside immunoglobulin loci,^76^, ^77^, ^78^ which targets highly transcribed genomic regions with specific sequence preferences.

Notably, Lamin B1 loss in vivo also led to the emergence of de novo DNA motifs enriched at DSB sites. These motifs corresponded to transcription factors involved in hematopoiesis and heterochromatin regulation, indicating that Lamin B1‐dependent chromatin architecture may limit DNA fragility by restricting transcription factor access to vulnerable genomic regions.

Finally, our patient data support the translational relevance of Lamin B1 loss in DLBCL. *LMNB1* expression was diminished in the N1 and EZB genetic subtypes, linking reduced Lamin B1 levels to specific molecular categories of disease. Moreover, the observation that *LMNB1* was lowest in stages II and III, but not further reduced in stage IV, within GCB‐DLBCL indicates that Lamin B1 loss does not simply track with clinical stage. Rather, it may mark a subtype‐specific state of nuclear and chromatin remodeling that is selected during disease evolution. The association between low *LMNB1* expression and shorter PFS, together with the finding that low Lamin B1 protein predicted adverse outcome specifically in late (≥24 months) patients, further suggests that Lamin B1 is more closely linked to long‐term disease behavior than to the determinants of early mortality. In this context, reduced Lamin B1 may define tumors with increased GI and adaptive fitness, thereby promoting persistence and relapse over time.

## MATERIALS AND METHODS

### Cell lines and Lamin B1 knockdown

Burkitt's lymphoma BL2, GCB‐DLBCL OCI‐LY8, and human embryonic kidney HEK293 cells were obtained from the German Collection of Microorganisms and Cell Culture (Deutsche Sammlung von Mikroorganismen und Zellkulturen, DSMZ). BL2 and OCI‐LY8 were cultured in RPMI‐1640 medium supplemented with l‐glutamine, 10% FBS, and 1% penicillin/streptomycin (PS). HEK293 cells were maintained in Dulbecco's Modified Eagle's medium supplemented with 10% FBS and 1% PS. The cell lines were maintained at 37°C with 5% CO_2_, passaged every 48 h, and routinely screened for mycoplasma contamination. To generate stable shRNA Lamin B1 knockdown cell lines, BL2 cells were transduced with lentiviral particles containing specific shRNA sequences (shLMNB1 #1: [V3SH11252‐224783461], shLMNB1 #2: [V3IHSMCG_8167334]). For validation, OCI‐LY8cells were transduced with shRNA (shLMNB1 #1: [V3SH11252‐224783461] against LMNB1 mRNA using a calcium phosphate transfection protocol. Briefly, HEK293 cells were transfected using a Dharmacon trans‐lentiviral shRNA packaging kit with calcium phosphate transfection reagents (TLP5913), according to the manufacturer's protocol. BL2 or OCI‐LY8 cells were transduced with lentivirus‐containing supernatant and positively selected with puromycin at a 2.5 µg/mL concentration for 72 h. Target cell lines containing lentiviral vectors were incubated with 500 ng/mL of doxycycline (DOX) to induce shRNA expression. The percentage of GFP‐expressing cells was assessed using flow cytometry 24 h after the addition of DOX, and Lamin B1 knockdown was assessed 72 h later. For transient Lamin B1 knockdown, cells were subjected to electroporation as previously described.^26^ Lamin B1 nuclear incorporation and total protein levels were assessed using IF and western blotting, respectively. Cell counts and viability were measured by acridine orange/propidium iodide staining using a Luna dual fluorescent cell counter (Logos Biosystems).

BL2 AID^−/−^ cells were kindly provided by Claude‐Agnes Reynaud (INSERM U1151) and were described previously.^79^ BL2 AID ^wt^ refers to the Burkitt's lymphoma BL2 obtained from the German Collection of Microorganisms and Cell Culture (Deutsche Sammlung von Mikroorganismen und Zellkulturen, DSMZ).

### Western blotting

Total cell lysates were prepared by lysing harvested cells in 2X NuPAGE LDS sample buffer with protease and phosphatase inhibitors. Lysates were then sonicated for 15 cycles (30 s on/off) using a Bioruptor Pico (B01060010). Denatured proteins were separated by electrophoresis and transferred onto methanol‐activated PVDF membranes. The membrane was then blocked with 3% BSA/TBS‐T for one hour and incubated with the primary antibody at the specified dilution and duration (see Resource Table). The membrane was washed with TBS‐T and incubated for one hour with a corresponding horseradish peroxidase (HRP)‐linked secondary antibody, anti‐rabbit IRDye 680RD secondary antibody, or anti‐mouse IRDye 800RD secondary antibody. Images were developed with Pierce ECL Substrate using the Amersham Imager 600RGB system or the Li‐Cor imaging system. The images were processed using Image Studio and ImageJ software.

### Single‐cell electrophoresis

The single‐cell electrophoresis (COMET) assay was performed according to the protocol from the Trevigen Comet Assay kit (4250‐050‐K) with minor modifications. Briefly, transduced or etoposide‐treated cells (positive control) were collected, washed with ice‐cold PBS (no Ca/Mg), and mixed with 1% low‐melting‐point molten agarose (37°C) in a 1:10 cell‐to‐agarose ratio. The cell suspension was spread evenly onto a pre‐chilled CometSlide and cooled at 4°C for at least 20 min in the dark before overnight immersion in a cold lysis solution. The slides were briefly washed with PBS and incubated in a freshly prepared alkaline solution at 4°C (pH >13) for an hour in the dark. Electrophoresis was carried out in a 4°C alkaline electrophoresis solution at 21 V for 20 min with a constant current of 300 mA. Immediately after electrophoresis, the slides were washed with dH_2_O for 5 min and immersed in 70% EtOH for an additional 5 min. The cells were brought to a single plane by air‐drying at 37°C for at least 15 min. SYBR Gold was used to stain DNA for 30 min in the dark. Slides were then air‐dried in the dark, and images were taken using a Nikon Ci‐L upright fluorescent microscope at ×20 magnification. TriTek CometScore 2.0, an automatic comet assay software (TriTek Corp.), was used to quantify tail moment in individual cells. Comets with a tail moment >250 were classified as apoptotic cells and were removed from quantification.

### Flow cytometry

To assess GFP expression, live cells were washed with PBS and incubated with DAPI (1 μg/mL) prior to flow cytometry analysis.

### WES and topology mapping

Transient Lamin B1 knockdown was performed as previously described.^26^ Whole exome sequencing (WES) with HiSeq. 2 × 150 bp, with an average sequencing depth of ×500 for BL2 AID^wt^, BL2 AID^−/−^, and PCL12^wt^ cells 48 h after transfection with siRNAs targeting Lamin B1. Raw reads were paired, and adapters were trimmed using fastp. Reads were mapped to the hg38 reference genome using BWA‐MEM. SNVs were called with Mutect2 (v3.5), and variants found specifically in siLMNB1‐treated samples were used as input for signature deconvolution using deconstructSigs. Mutational clustering was performed using KaryoplotR (v1.22.0) package, and kataegis foci were detected using katdetectr (v1.12.0).

### RNA isolation and sequencing

RNA was extracted using the Qiagen RNeasy Kit according to the manufacturer's protocol. BL2 cells were harvested for RNA extraction and library preparation after 72 h of shLMNB1 treatment. Paired‐end sequencing was performed using Illumina HiSeq. 2500 with a minimum of 30 million reads per sample. Briefly, adapter sequences were trimmed using TrimGalore (v0.6.3) and aligned to hg19 using HISAT2 (v2.0). QualiMap (v2.2.2) was used to assess the quality of alignment, and raw read counts were quantified using FeatureCounts (v1.6.4) and used for downstream analysis.

### Differential gene expression analysis

Differential gene expression analysis was performed using the DESeq. 2 (v1.28.1) package in R. Significantly differentially expressed genes (DEG) were filtered based on a *p*‐adjusted (padj) < 0.05, and a log2 fold change (log2FC) greater than 0.50 or less than −0.50.

### Deletion of Lamin B1 in mouse splenic GC B cells

All mice were on a C57BL/6 background using the *Cre*/*loxP* system, which engages in the *Cre*‐mediated deletion of *LMNB1*. GC‐targeted *LMNB1* deletion was accomplished by crossing the C57BL/6N‐Lmnb1^tm1c(EUCOMM)Wtsi^/WtsiH (referred to as *Cγ1cre* ^+/−^; *Lmnb1* ^*fl/fl*^) strain, obtained from EMMA (EM:10615), and B6.129P2(Cg)‐*Ighg1* ^*tm1(cre)Cgn*^/J (referred to as *Cγ1cre* ^+/−^), kindly provided by Dr. Dinis Calado (Crick Institute, UK).^80^ *Cre* recombinase activation was induced by the stimulation of the immune response after an intraperitoneal (IP) injection of 100 μL ImjectTM Alum Adjuvant with 100 µg of NP‐CGG (Chicken Gamma Globulin) Ratio 10‐19 (N‐5055B‐1, 2 B Scientific) at a concentration of 0.5 mg/mL. *Cγ1‐Cre* mice were used as heterozygous for the *Ighg1*<tm1(cre)Cgn> gene, and the LaminB1^fl/fl^ mouse strain was homozygous for Lmnb1^tm1a(EUCOMM)^/WtsiH. All control and experimental mice were randomized among littermates (8–12 weeks old), with no sex bias between groups. All experiments involving mice were performed under the Queen Mary of London Veterinary Oversight with the UK Home Office authorization.

### Immunohistochemistry

Paraffin‐embedded samples were dewaxed twice in 100% xylene for 3 min, followed by incubation in 100% ethanol for 3 min, and twice in 100% ethanol (3% H_2_O_2_) for 3 min to block endogenous peroxidases. Prior to the antigen retrieval step, the samples were successively placed in 100% ethanol and deionized H_2_O for 3 min. Antigen retrieval was performed by boiling the samples at the maximum temperature in a pressure cooker in the presence of 1× antigen unmasking solution for 10 min. Slides were then placed in 1× DAKO wash buffer and incubated with primary and secondary antibodies, followed by the VIP HRP Detection Kit (see Resource Table). Hematoxylin was used as the nuclear stain. The dehydrated slides were mounted and scanned with a digital slide scanner (NanoZoomer S210, Hamamatsu C13239‐01) at ×20 original magnification. For multiplex immunohistochemistry, the same mouse tissue sections were subjected to sequential rounds of staining by stripping the previous staining and repeating the protocol described above with each subsequent antibody. At least four randomly selected GCs per spleen from a minimum of five mice were selected and used for the analysis. Raw images were processed and analyzed using QuPath (v0.2.0) and Fiji (v2.0.0).

### IF

For IF imaging, cells were washed with ice‐cold phosphate‐buffered saline (PBS) and spun onto a poly‐l‐lysine‐coated slide for 5 min in a Shandon Cytospin centrifuge (#22004). Immediately after centrifugation, the cells were fixed in 4% paraformaldehyde (PFA) for 15 min and permeabilized with 0.2% Triton‐X/PBS. Slides were then blocked with 3% bovine serum albumin (BSA)/PBS‐T (0.2% Tween‐20) for one hour and incubated with the primary antibody at specifically optimized concentrations and time durations according to the Resource Table. Slides were then washed with PBS‐T and incubated with secondary fluorescently conjugated antibodies and DAPI at a concentration of 1 µg/mL for 45 min in the dark. Slides were mounted using ProLong Gold, and images were taken with a Nikon Ci‐L fluorescent microscope or a confocal Zeiss LSM550/LSM710 microscope. Raw images were analyzed using ImageJ software for the quantification of γ‐H2AX, and 53BP1 foci were counted using the MATLAB FoCo script. The color balance was adjusted using the same settings across all experimental conditions.

### In‐suspension breaks labeling in situ and sequencing (sBLISS)

To generate the genome‐wide DSB profile, the sBLISS methodology was adapted in BL2 cells (control and shLMNB1) and FACS‐sorted mouse splenocytes (GC B cells and Naïve B cells) after shLMNB1‐mediated depletion and immunization, respectively. BL2 cells were used in triplicate, and mouse splenocytes were obtained from a minimum of four mice (*Cγ1Cre* ^+/−^ and *Cγ1Cre* ^+/−^; *Lmnb1* ^*fl/fl*^) from two independent immunizations with 100 μg NP‐CGG for ten days. Prior to cell sorting, we enriched the GC B cell population using the Germinal Center B Cell MicroBead Kit, according to the manufacturer's protocol. The sBLISS template was generated as previously described.^48^ Libraries were analyzed using the DNA Tapestation 4200 and sequenced as single‐end (1 × 75 bp) reads on the NextSeq platform.

### Preprocessing of sequencing data

Data analysis was performed using a publicly available script created specifically for the sBLISS analysis. Raw FASTQ files were merged, and reads were filtered based on the associated sample barcode (see Resource Table), allowing up to 1 mismatch in the barcode sequence. Prefixes in each read were trimmed, and sequences were aligned to the hg19 or mm10 reference genome using BWA‐MEM (v0.7.17) and bedtools (v2.30.0). Aligned reads with a mapping quality score ≤60 and PCR duplicates marked by SAM tools (v1.9) were filtered out. The generated BED files containing unique DSB locations were used for downstream analysis.

## HOTSPOT DETECTION

The detection of sBLISS hotspots was performed using Macs2 (v2.1) to call peaks from the BED files of UMI‐DSBs (no background model, no cross‐correlation between strands around the hotspots, shift reads by −100 bp, and extend by 200 bp). DSB hotspots were defined as the peaks identified by Macs2 with a *q* < 0.05, fold enrichment >4, and pileup >10. Circus plots were used to visualize the unique sBLISS hotspots per sample.

## GENOMIC REGION DEFINITIONS

We constructed strand‐agnostic genomic intervals for the following categories: TSS, Promoter, 5′‐UTR, 3′‐UTR, CDS, Exon, and Intergenic. TSS windows were defined as ±250 bp around annotated transcript start sites (upstream = 250 bp; downstream = 250 bp). Promoters were defined as −2000 to +500 bp relative to the annotated transcript start site (upstream = 2000 bp; downstream = 500 bp). CDS and UTR intervals were taken directly from TxDb transcript annotations. To avoid double‐counting between exon‐derived categories, the Exon category was defined as exonic sequence not overlapping CDS or UTR intervals (i.e., Exon = Exons\(CDS ∪ 5′‐UTR ∪ 3′‐UTR)). Intergenic regions were defined as the complement of gene bodies (genes from TxDb) across standard chromosomes (i.e., genome\genes). All feature sets were reduced (merged) to remove overlaps within each category.

Each hotspot peak was represented by a single coordinate (peak center) and assigned to one category using a fixed precedence scheme to enforce mutual exclusivity and prevent counting the same hotspot in multiple overlapping annotations. Precedence was applied in the following order: CDS → 5′‐UTR → 3′‐UTR → TSS → Promoter → Exon → Intergenic. Hotspots overlapping multiple annotations were assigned to the highest‐precedence category. This yielded a single region label per hotspot for downstream counting and enrichment analyses.

### Observed/Expected (O/E) using Control as expected baseline

To quantify region‐specific enrichment of DSB hotspots in LMNB1‐deficient cells relative to the Control baseline, we computed an Observed/Expected (O/E) ratio for each genomic region. After exclusive region assignment (above), we counted hotspots per region for Control ($C_{r}$) and LMNB1‐deficient cells ($O_{r}$), where rdenotes the region category.

Expected counts for LMNB1‐deficient cells were derived from the Control regional composition. Control region probabilities were calculated as:

$$p_{r}=\frac{C_{r}}{\sum_{r}C_{r}},$$

and the expected number of LMNB1‐deficient hotspots in region r, under the null hypothesis that the LMNB1‐deficient distribution follows the Control distribution, was:

$$E_{r}=p_{r}\times \sum_{r}O_{r}.$$

The Observed/Expected ratio was then computed as:

$${O/E}_{r}=\frac{O_{r}+\epsilon }{E_{r}+\epsilon },$$

where ϵ is a small pseudocount (0.5) to stabilize estimates in categories with low expected counts. For visualization, we reported $\log_{2}({O/E}_{r})$; values >0 indicate enrichment and values <0 indicate depletion in LMNB1‐deficient cells relative to the Control baseline.

This approach tests redistribution across regions in LMNB1‐deficient cells relative to the empirical control composition.

### sBLISS and RNA‐seq data integration

Significant DEGs from the shLMNB1 knockdown analysis (adjusted P < 0.05, log₂FoldChange > 1) were annotated with genomic coordinates using the EnsDb.Hsapiens.v75 using R package. Both the DEG genes and the sBLISS DSB sites were converted to GRanges objects, harmonized to the hg19 reference genome, and analyzed for overlap.

## LAD DEFINITIONS

### BL2

Raw paired‐end FASTQ files from GSE89869 (SRR5022916 and SRR5022918) were processed using standard QC and preprocessing. Read quality was assessed using FastQC, followed by adapter/quality trimming using fastp, and FastQC was repeated on trimmed reads. Trimmed reads were aligned to the UCSC hg38 reference genome with Bowtie2, and alignments were processed using samtools to generate coordinate‐sorted BAMs, retain properly paired high‐confidence reads (MAPQ ≥ 30), and remove PCR duplicates using a fixmate/markdup workflow. Deduplicated BAMs from the two biological replicates were merged to generate a combined Lamin B1 ChIP‐Seq alignment for downstream domain calling. LMNB1‐enriched domains were defined using a bin‐based approach (100 kb bins). sBLISS‐derived LMNB1‐associated DSB hotspots were originally called on the hg19/GRCh37 assembly and were therefore converted to hg38 coordinates using UCSC liftOver (hg19 → hg38 chain); unmapped hotspots were retained as a separate output and excluded from overlap analyses. The fraction of DSB hotspots overlapping bin‐derived LMNB1 domains was then quantified as “inside” versus “outside” LMNB1‐enriched regions.

### Mouse

LAD calls from GSE17051, GSE156293, and GSE241483 were harmonized to mm10 and represented on a common 10 kb genomic bin grid. Pairwise concordance was quantified by overlap with the GSE17051 cLAD set using the Jaccard index and base‐pair overlap, yielding Jaccard values of 0.486155 (GSE156293 vs. GSE17051) and 0.313103 (GSE241483 vs. GSE17051), with 58.44% and 51.96% of GSE17051 LAD base pairs overlapping GSE156293 and GSE241483, respectively. cLADs were defined as 10 kb bins present in all three datasets, and fLADs as bins present in a subset of datasets.

### LAD annotation and hotspot overlap (mm10)

LAD coordinates (mm10) were compiled from the three Lamin B1 LAD datasets (GSE17051, GSE156293, GSE241483). LAD intervals were filtered to chromosomes present in the mm10 UCSC chrom.sizes file, chromosome naming was harmonized (e.g., 1 → chr1), and overlapping/adjacent LAD segments were merged (bedtools merge) prior to coverage calculations. Genomic LAD coverage was computed as total merged LAD base pairs divided by total genome length from chrom.sizes. Hotspot overlap with LADs was quantified as the number of hotspot intervals with any intersection with LADs (bedtools intersect ‐u).

### DSB quantification at the gene level

DSB burden per gene was quantified from sBLISS readouts using BED/BED.GZ files. Break coordinates were imported into R as GRanges objects (rtracklayer/GenomicRanges) and harmonized to hg19 UCSC chromosome naming, retaining standard autosomes and sex chromosomes. Gene bodies were defined using hg19 gene annotations from TxDb.Hsapiens.UCSC.hg19.knownGene, and input driver gene lists for CLL^50^ and DLBCL^81^ were mapped from symbols to Entrez IDs (org.Hs.eg.db; SYMBOL/ALIAS) to retrieve genomic coordinates. For each sample, overlaps between DSB sites and gene bodies were identified, and DSB counts were summed across all break sites falling within each gene, with genes lacking overlaps retained as zero.

For percentage‐based summaries, driver genes were classified as DSB‐positive if they overlapped ≥1 detected DSB within the gene body in the shLMNB1 condition, with the full curated driver gene list used as the denominator. To distinguish clustered break regions from single events, DSB hotspots were defined as reproducibly enriched regions called with MACS2 (*q* < 0.05, fold enrichment >4, pileup >10); genes were classified as hotspot‐positive if they overlapped at least one hotspot.

## DISCOVERY OF DE NOVO MOTIFS AND ANALYSIS

To identify DNA de novo motifs enriched around Lamin B1‐associated DSB hotspots, we used MEME Suite (v5.5.8). Sequences ±100 bp flanking the summit of high‐confidence DSB peaks were extracted and analyzed with motif widths constrained between 6 and 12 bp, and the top 5 motifs were retained. The top five most significant motifs identified by MEME were used for further analysis. The R packages universalmotif, ChIPseeker, and GenomicRanges were employed to scan for all motif occurrences within the identified peak regions of the *Mus musculus* genome (mm10 assembly). Genomic features (3′‐UTR, 5′‐UTR, CDS, exons, intergenic regions, introns, promoters, and TSS) were hierarchically annotated, and associated genes were identified. To identify biological processes potentially affected by motif‐associated DNA damage, we extended the genomic coordinates of de novo discovered motifs by ±3 kb and mapped them to the nearest TSS. This yielded a list of genes located in proximity to the motif hotspots. ClusterProfiler was used to perform a GO enrichment analysis on the associated genes to infer the biological pathways linked to the discovered motifs.

The de novo motifs were also matched to known transcription factors (TF) motifs using Tomtom (MEME Suite) with the following parameters: ‐verbosity 1 ‐min‐overlap 5 ‐mi 1 ‐dist pearson ‐evalue ‐thresh 10.0 ‐time 300. We queried against two motif collections: the Uniprobe mouse database and the JASPAR 2024 CORE vertebrates non‐redundant database. Matches were ranked by Pearson correlation and minimum information content overlap of at least five positions. To contextualize the biological relevance of matched TFs, we utilized the CistromeDB, querying publicly available ChIP‐seq data from different cell types to evaluate TF binding site enrichment near our input regions. GIGGLE similarity scores were computed, and enrichment analysis against reference epigenomic datasets was performed.

### Clinicopathological analysis of *LMNB1* expression and GEP in CLL and DLBCL

For CLL gene expression analysis relative to LMNB1 expression, we downloaded a dataset from patients with CLL (REACH trial, GEO: GSE52811). Normalization, cluster analysis, and subtype classification were conducted as previously described.^43^ *LMNB1* expression was assessed in the GI subtype (highest frequency of *TP53* alterations, shortest PFS in *TP53* WT cases), in comparison to the (I)EMT‐L subtype (lowest frequency of *TP53* alterations, most prolonged PFS). For DLBCL, we utilized a publicly available dataset from TCGA (phs001444) to assess *LMNB1* expression and the clinical outcomes of 481 DLBCL patients.^63^ Raw count files and clinical data were obtained from TCGA and the previously published DLBCL study,^63^ respectively. Log2‐transformed counts per million (Log2CPM) were used for GEP and clinical evaluation of *LMNB1* expression. Kaplan−Meier survival analysis for PFS was conducted based on *LMNB1* expression levels in DLBCL patients. The group cutoff was set based on the quartile or median *LMNB1* expression.

In the DLBCL validation cohort, Lamin B1 protein expression was assessed in previously reported tissue microarrays^64^ by immunohistochemistry using the protocol described above. For each patient, a mean Lamin B1 *H*‐score was derived from three replicate samples. Patients were initially stratified according to survival of less than or greater than 2 years. Within each survival group, cases were further dichotomized using the mean Lamin B1 *H*‐score as the cutoff, with patients classified as Lamin B1 high or Lamin B1 low. Kaplan–Meier curves were then generated to evaluate differences in survival between groups.

Ethical approval was obtained from the East London & The City Health Authority Local Research Ethics Committee (Reference number 10/H0704/65), and written informed consent was obtained in accordance with the Declaration of Helsinki.

### Study approval

All animal studies were conducted in compliance with the UK Home Office‐approved licenses (Animals (Scientific Procedures) Act 1986 and the EU Directive 2010). Animals were maintained in local facilities, and experiments were approved by the local ethical committees under Home Office license P68650650.

### Statistical analysis

Generated data were obtained from independent biological replicates, as indicated in the figure legend or the corresponding methods section. For IHC, IF, and western blotting analysis, raw images were processed using Fiji and QuPath. Foci counting was performed using the Fiji or FociCounter algorithm in MATLAB (MathWorks). Survival analysis was performed in R using the ggsurvplot package. Numerical data were analyzed and plotted in Prism v8.3 (GraphPad Software, Inc.) or R. P‐values were calculated using a two‐tailed unpaired *t*‐test unless stated otherwise.

## AUTHOR CONTRIBUTIONS

**Filip Filipsky**: Conceptualization; methodology; formal analysis; investigation; writing—review and editing; writing—original draft. **Katarina B. Chapman**: Writing—review and editing. **Johannes Bloehdorn**: Investigation; formal analysis. **Oscar Maiques**: Formal analysis; data curation. **Abigail Lee**: Data curation; resources. **Andrew Clear**: Resources. **Jun Wang**: Investigation; software; formal analysis. **John Gribben**: Investigation; writing—review and editing; resources. **Michael Hausmann**: Writing—review and editing; resources. **Christoph Cremer**: Resources. **Andrejs Braun**: Conceptualization; methodology; funding acquisition; resources; supervision. **Marta C. Sallan**: Conceptualization; methodology; software; formal analysis; writing—original draft; writing—review and editing; supervision; investigation. **Tanya Klymenko**: Conceptualization; writing—review and editing; supervision.

## CONFLICT OF INTEREST STATEMENT

The authors declare no conflict of interest.

## FUNDING

This study was supported by Blood Cancer UK Project 19001 “Lamin B1‐mediated genomic instability in leukemia and lymphoma” and Cancer Research UK Centre City of London Centre grant C7893/A26233. O.M. is supported by Barts Charity (MGU0504).

## Supporting information


**Supplementary Figure 1. Decreased Lamin B1 is associated with CLL GI and mutagenesis in malignant B cells**. **(A)** Heatmap showing gene expression profile of DNA mismatch repair (MMR)‐associated genes in GI and (I)EMT‐L CLL subtypes (*n* = 173). **(B)** Schematic representation of the WES experiment to deconvolute the mutational pattern in siLMNB1 lymphoma cells. **(C)** Representative analysis of single base substitutions (SBS) detected in Lamin B1 knockdown cell lines (BL2 AID^‐/‐^BL2 AID^wt^, PCL12) after comparing with control, confirming the enrichment in C > T substitution.
**Supplementary Figure 2. In vitro Lamin B1 reduction translates into increased GI, unrelated to** increased **expression of AICDA or APOBEC genes**. **(A)** Flow cytometry analysis of GFP expression following shLMNB1 induction in BL2 cells. **(B)** Flow cytometry analysis of GFP expression following shLMNB1 induction in OCI‐LY8 cells. **(C)** Western blot showing Lamin B1 expression in control and shLMNB1‐expressing BL2 cells. **(D)** Western blot confirming reduced Lamin B1 expression in RNA‐seq replicates R1 and R2. **(E)** Normalized RNA‐seq expression levels of LMNA, LMNB1, and LMNB2 comparing control and shLMNB1 conditions. **(F)** Barplot depicting expression levels of AICDA and APOBEC family members in control versus shLMNB1 conditions, derived from RNA‐seq data. **(G)** Western blot showing the expression of 53BP1 and Lamin B1 in OCI‐LY8 cells across three replicates. **(H)** Western blot analysis of Lamin B1 and phospho‐γH2AX levels in OCI‐LY8 cells following shLMNB1. (**I**) Western blot showing Lamin B1 downregulation upon shLMNB1 from the same experiment presented in OCI‐LY8 cells in (J). (**J**) Representative immunofluorescence images of 53BP1 (red) and γH2AX (green) in control and shLMNB1 72 h post‐doxycycline induction in OCI‐LY8 cells. (**K**) Foci quantification of 53BP1 (red) and γH2AX (green) and colocalization (yellow) in control and shLMNB1 in OCI‐LY8 cells. **(L)** Representative immunofluorescence images of γH2AX (green) and Lamin B1 (red) in PNA⁻ and PNA⁺ cells isolated from *Cγ1Cre* ^*⁺/⁻*^ and *Cγ1Cre* ^*⁺/⁻*^ *; Lmnb1* ^*fl/fl*^ mice.
**Supplementary Figure 3. Lamin B1 depletion in GC B cells results in GC phenotypic changes**. (**A**) Representative images of BCL6 immunohistochemistry on *Cγ1Cre* ^*+/‐*^ and *Cγ1Cre* ^*+/‐*^ *;Lmnb1* ^*fl/fl*^ spleens 21 days post immunization. Cell detections (Red = positive BCL6 staining; blue = negative BCL6 staining) in GCs. (**B**) Barplot showing GC number measured by BCL6+ clusters ( > 10 cells) per spleen section. Comparison of *Cγ1Cre* ^*+/‐*^ (n = 4 mice) and *Cγ1Cre* ^*+/‐*^ *;Lmnb1* ^*fl/fl*^ (n = 4 mice) in at least two independent immunizations. Two‐tailed t‐test (p‐value < 0.01). (**C**) Barplot showing GC area measured using BCL6+ clusters ( > 10 cells) per spleen section. Comparison *Cy1Cre* ^*+/‐*^ *;Lmnb1* ^*fl/fl*^ (n = 4 mice) and *Cγ1Cre* ^*+/‐*^ (n = 4 mice) in at least two independent immunizations. Two‐tailed t‐test (p‐value < 0.01).
**Supplementary Figure 4. DNA break profiling reveals that** ***LMNB1*** **reduction induces genome‐wide genomic instability**. (**A**) Schematic overview of the bioinformatic pipeline for sBLISS data analysis. **(B)** Western blot showing Lamin B1 and phospho‐γH2AX expression levels of cells used for the sBLISS experiment. **(C‐D)** PCA plot of genome‐wide DSB distribution (1 Mb Bins) in (**C**) human BL2 samples and (**D**) mouse samples. Heatmap displaying the DSB burden per genomic region in (**E**) BL2 cells and (**F**) *in vivo* GC B cells and non‐GC B cells across experimental conditions. (**G**) Histogram showing the distribution of hotspot signal values in human shLMNB1 and mouse LMNB1 knockdown GC B cells.
**Supplementary figure 5. Lamin B1 associates DSB hotspots with chromatin regulators**. **(A)** AID hotspots (WRC, WRCY, and WGCW) location probability associated with DSBs in Naïve B cells compared to *Cγ1cre* ^*+/‐*^ *;Lmnb1* ^*fl/fl*^ GC B cells. (**B**) Top 4 and 5 enriched *de novo* motifs in *Cγ1cre* ^*+/‐*^ *; Lmnb1* ^*fl/fl*^ GC B cells. (**C‐D**) Cistrome analysis with GIGGLE scoring quantified the association between unique DSB hotspots, transcription factors (**C**), and histone modifications (**D**) in *Cγ1Cre⁺/⁻;Lmnb1* ^*fl/fl*^ mice. (**E**) Overlap of LMNB1‐depletion‐specific DSB hotspots in GC B cells with histone modification peaks from the GSE143293 ChIP–seq dataset.
**Supplementary figure 6. Decreased LMNB1 expression is associated with poorer clinical outcomes in patients with DLBCL.** (**A‐B**) Boxplot showing *LMNB1* expression across diagnostic stages in (**A**) ABC DLBCL subtypes (N = 205) and (**B**) GCB DLBCL subtypes (N = 126). (**C**) Kaplan–Meier analysis of early ( < 24 months) overall survival in patients, dichotomized by mean Lamin B1 H‐score. (**D**) Kaplan–Meier analysis of overall survival in late ( ≥ 24 months), dichotomized by the mean Lamin B1 H‐score.

supporting file 2.

## Data Availability

The data that support the findings of this study are available from the corresponding author upon reasonable request.
