# Supplementary material for: Lamin B1 safeguards the B cell genome and shapes lymphoma outcome
Source: Hemasphere. 2026 Jun 7;10(6):e70387. doi: 10.1002/hem3.70387 (PMC13242629; doi:10.1002/hem3.70387)

Supplementary Figure 1.

A

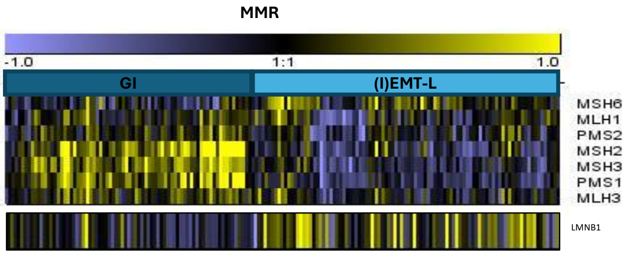

B

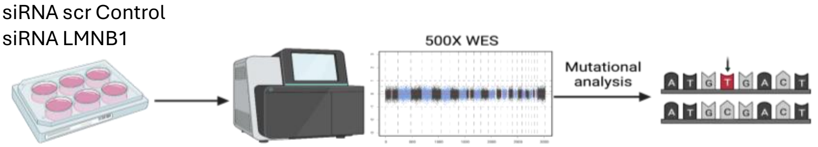

C

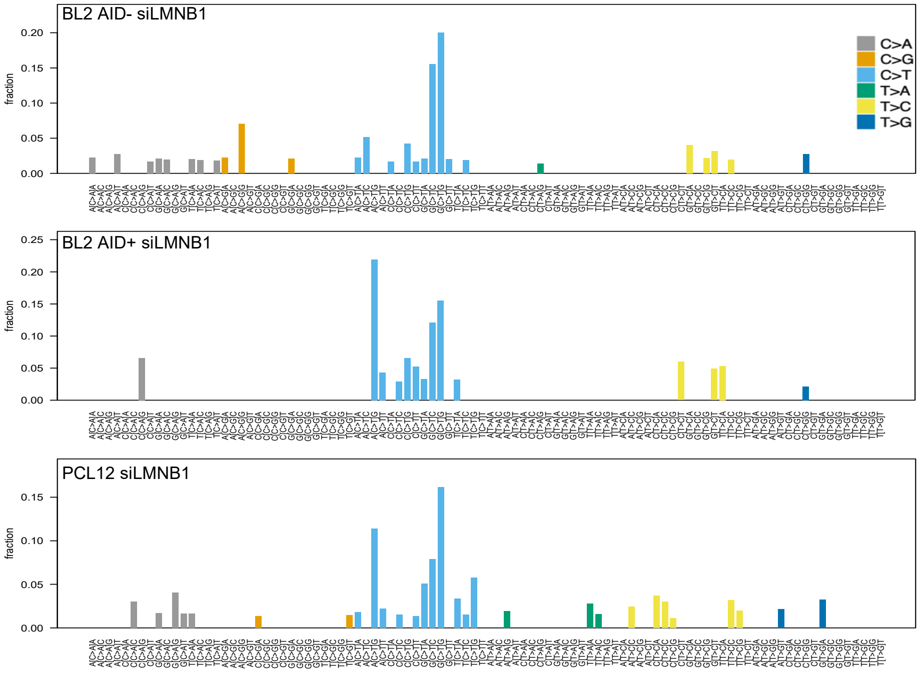

Supplementary Figure 2.

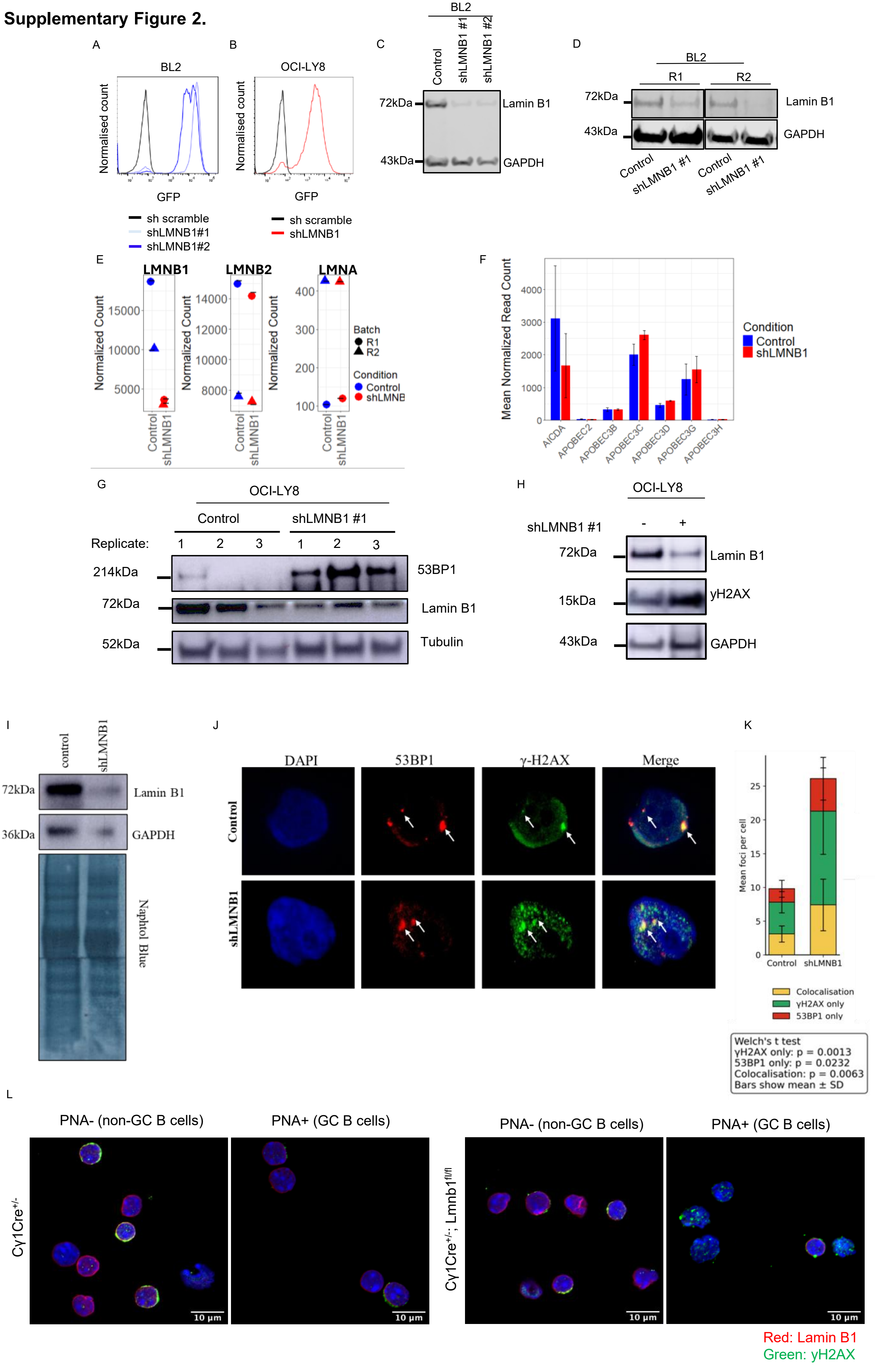

Supplementary Figure 3.

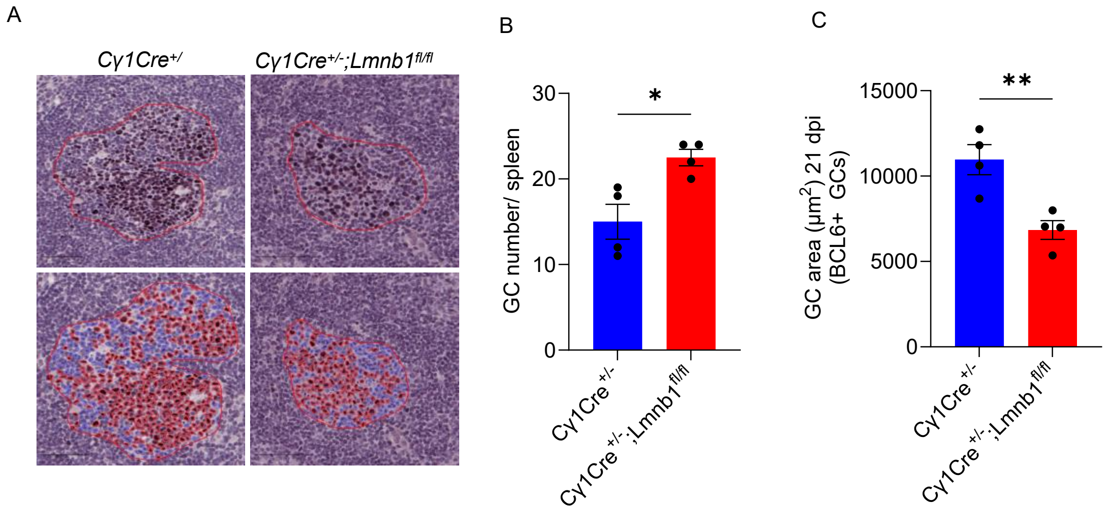

Supplementary Figure 4.

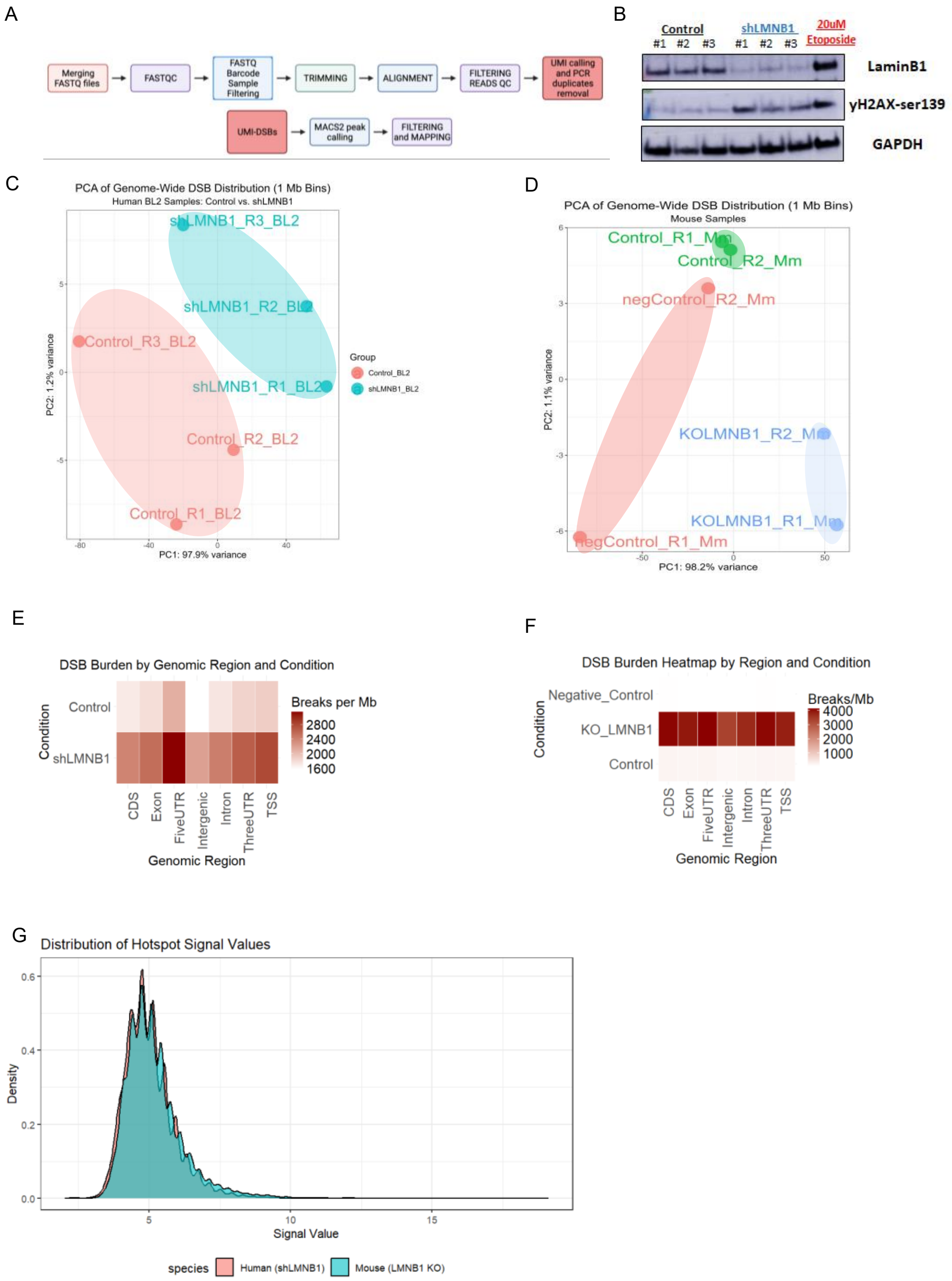

Supplementary Figure 5.

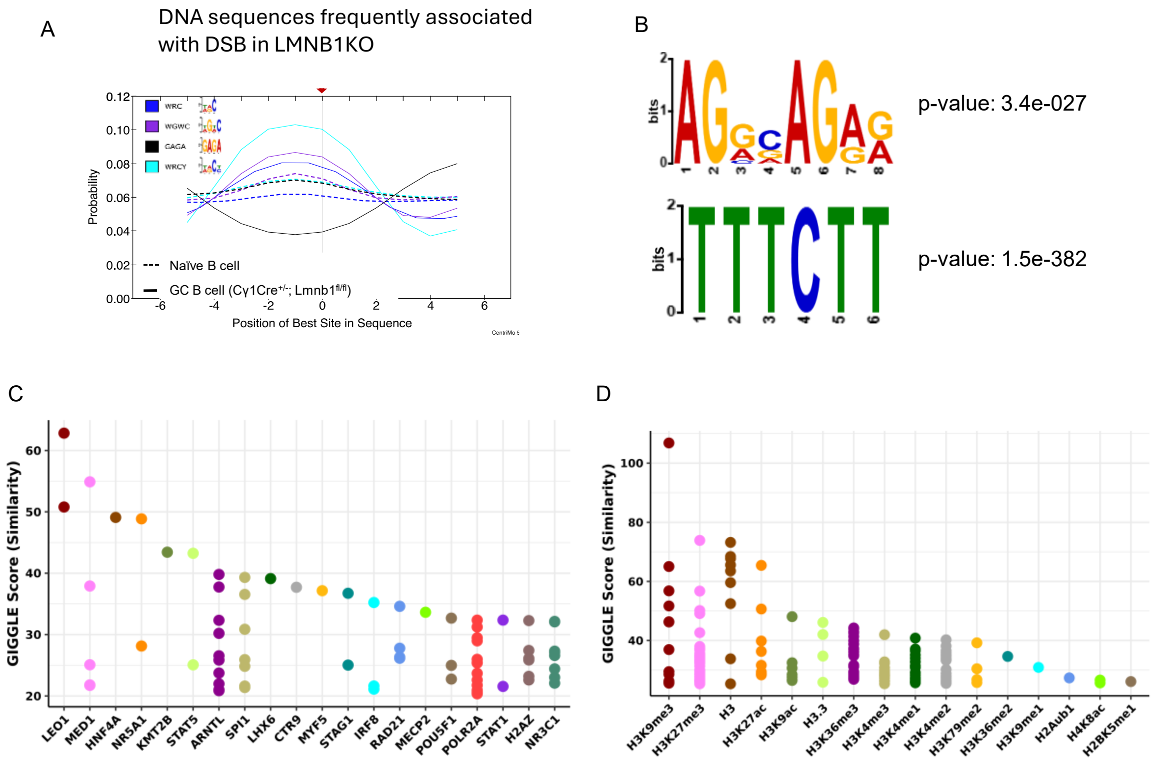

E

Histone mark ChIP-seq GSE143293	Unique hotspots/ total hotspots In LMNB1 depleted GC B cells
H3K27me3	4,314 of 24,810 hotspots (17.39%)
H3K27ac	39 of 24,810 hotspots (0.16%)

Supplementary figure 6.

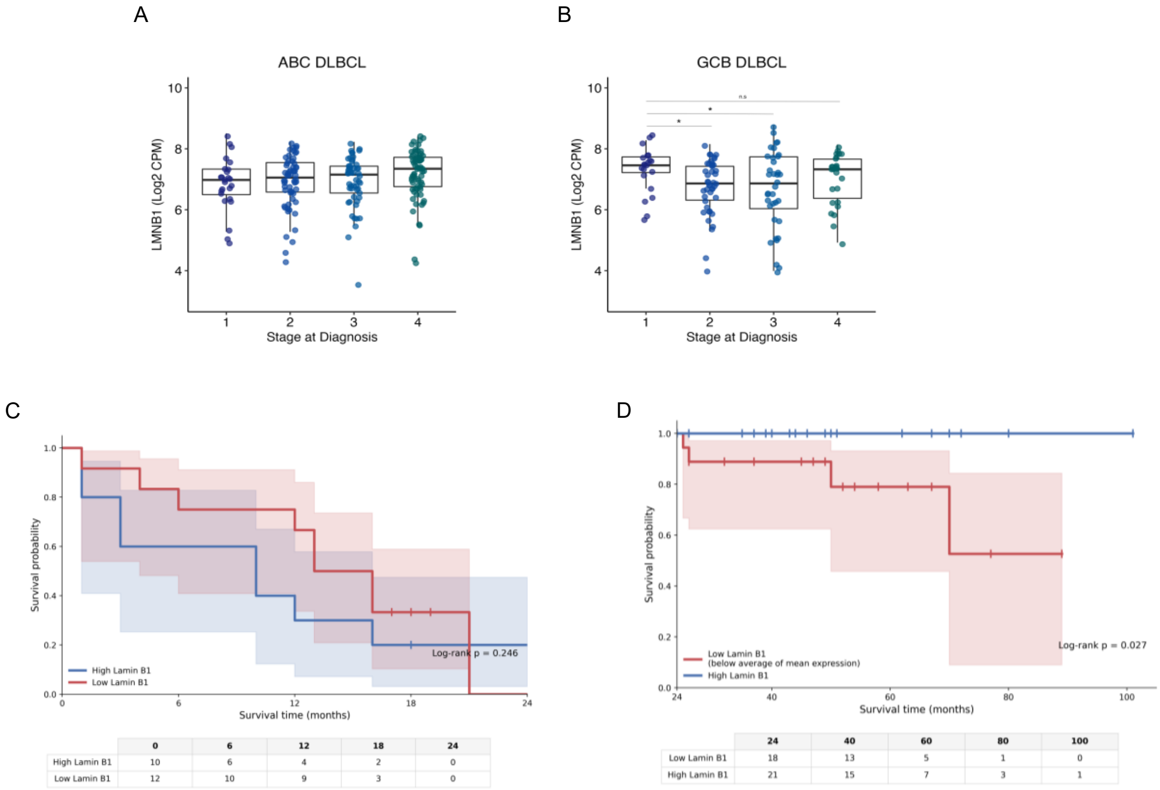

Supplement: Supplementary file 1 — Supplementary Figure 1. Decreased Lamin B1 is associated with CLL GI and mutagenesis in malignant B cells. (A) Heatmap showing gene expression profile of DNA mismatch repair (MMR)‐associated genes in GI and (I)EMT‐L CLL subtypes (n = 173). (B) Schematic representation of the WES experiment to deconvolute the mutational pattern in siLMNB1 lymphoma cells. (C) Representative analysis of single base substitutions (SBS) detected in Lamin B1 knockdown cell lines (BL2 AID‐/‐BL2 AIDwt, PCL12) after comparing with control, confirming the enrichment in C > T substitution. Supplementary Figure 2. In vitro Lamin B1 reduction translates into increased GI, unrelated to increased expression of AICDA or APOBEC genes. (A) Flow cytometry analysis of GFP expression following shLMNB1 induction in BL2 cells. (B) Flow cytometry analysis of GFP expression following shLMNB1 induction in OCI‐LY8 cells. (C) Western blot showing Lamin B1 expression in control and shLMNB1‐expressing BL2 cells. (D) Western blot confirming reduced Lamin B1 expression in RNA‐seq replicates R1 and R2. (E) Normalized RNA‐seq expression levels of LMNA, LMNB1, and LMNB2 comparing control and shLMNB1 conditions. (F) Barplot depicting expression levels of AICDA and APOBEC family members in control versus shLMNB1 conditions, derived from RNA‐seq data. (G) Western blot showing the expression of 53BP1 and Lamin B1 in OCI‐LY8 cells across three replicates. (H) Western blot analysis of Lamin B1 and phospho‐γH2AX levels in OCI‐LY8 cells following shLMNB1. (I) Western blot showing Lamin B1 downregulation upon shLMNB1 from the same experiment presented in OCI‐LY8 cells in (J). (J) Representative immunofluorescence images of 53BP1 (red) and γH2AX (green) in control and shLMNB1 72 h post‐doxycycline induction in OCI‐LY8 cells. (K) Foci quantification of 53BP1 (red) and γH2AX (green) and colocalization (yellow) in control and shLMNB1 in OCI‐LY8 cells. (L) Representative immunofluorescence images of γH2AX (green) and Lamin B1 (red [file HEM3-10-e70387-s001.pdf]
